# Supplementary material for: Screening a library of antibacterial compounds leads to discovery of novel inhibitors for Neisseria gonorrhoeae and Chlamydia trachomatis
Source: PLoS One. 2026 Feb 6;21(2):e0340486. doi: 10.1371/journal.pone.0340486 (PMC12880645; doi:10.1371/journal.pone.0340486)
Supplement: S1 Table — (DOCX) [file pone.0340486.s001.docx]

**Table S1: The positive hit compounds against *N. gonorrhoeae* FA1090.**

| Number | Drug name (Class) | Drug structure | Reference |
| --- | --- | --- | --- |
|  | Sulopenem (Beta-lactam) | 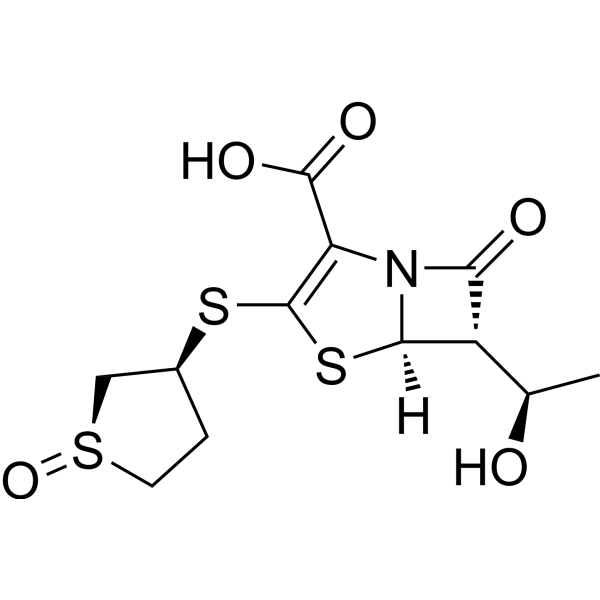 | [1] |
|  | Tebipenem  (Beta-lactam) | 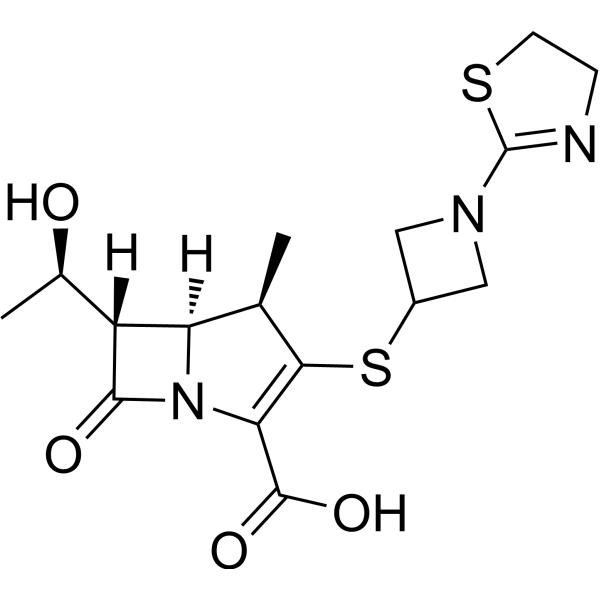 | [2] |
|  | Cefoselis sulfate  (Beta-lactam) | 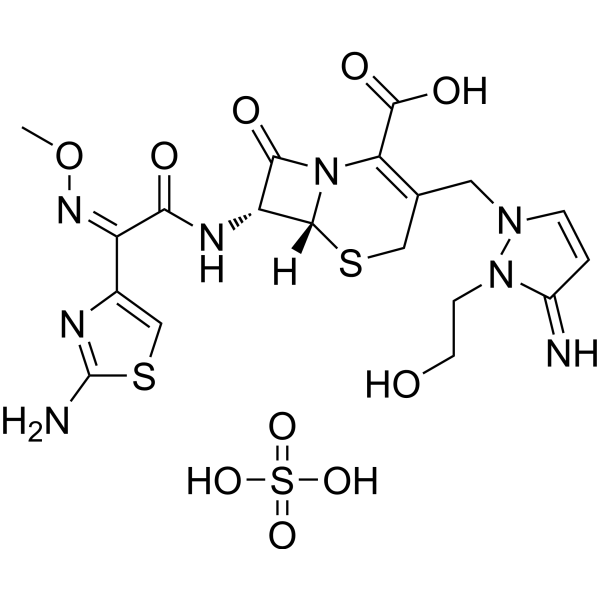 | [3] |
|  | Ceftobiprole medocaril  (Beta-lactam) | 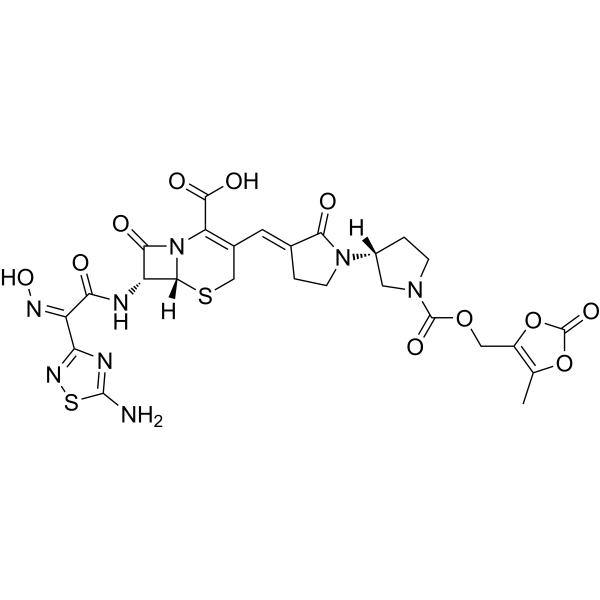 | [4] |
|  | ceftaroline or T-91825  (Beta-lactam) | 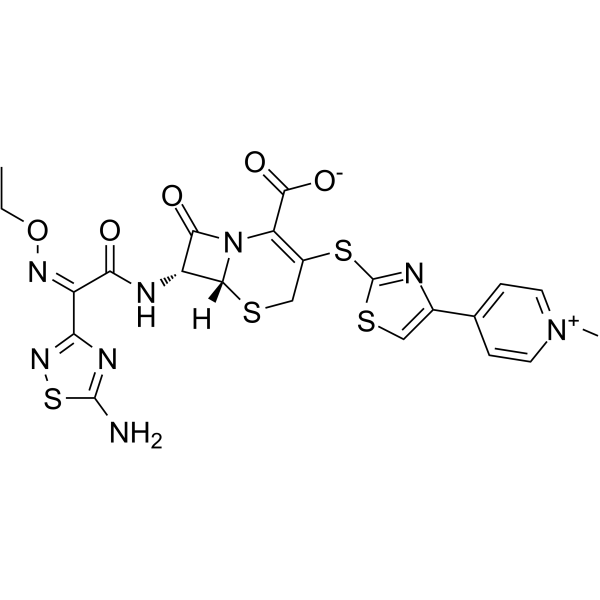 | [5] |
|  | **Cefotetan** (Beta-lactam) | 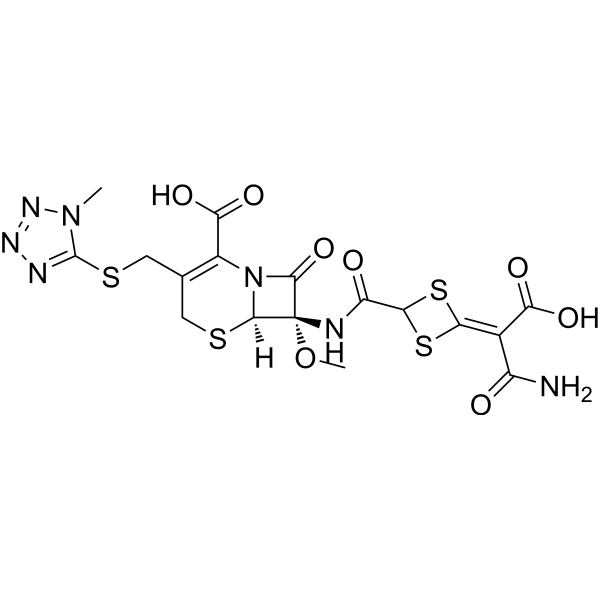 | [6] |
|  | Cefoperazone  (Beta-lactam) | 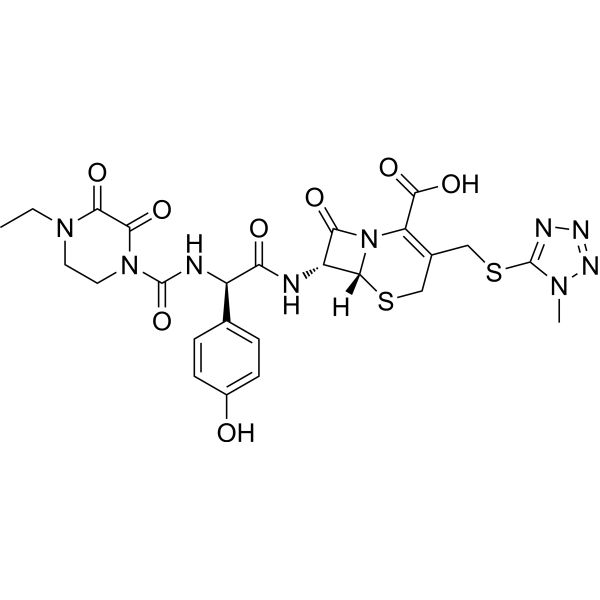 | [7] |
|  | **Cefetamet** (Beta-lactam) | 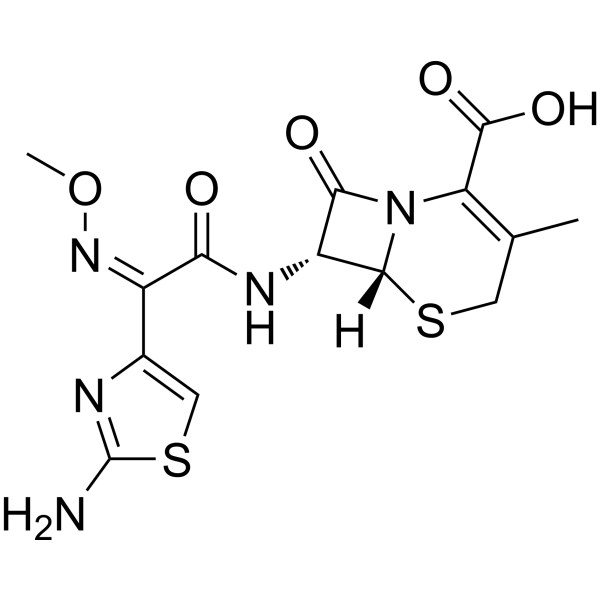 | [8] |
|  | **Cefditoren Pivoxil** (Beta-lactam) | 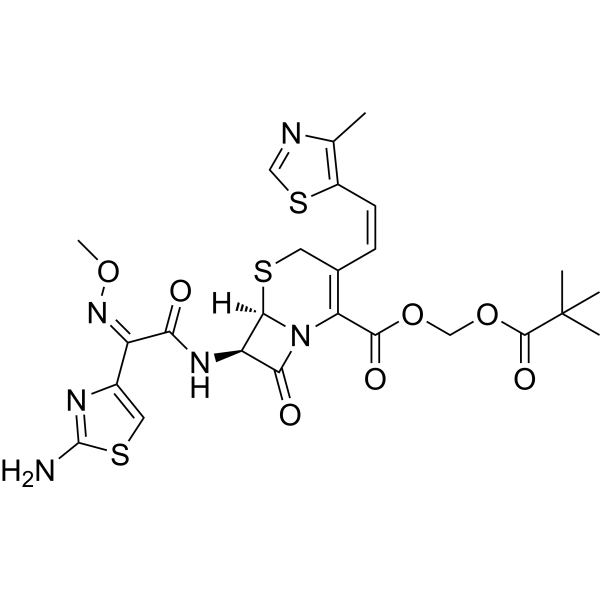 | [9] |
|  | **Cefotaxime** (Beta-lactam) | 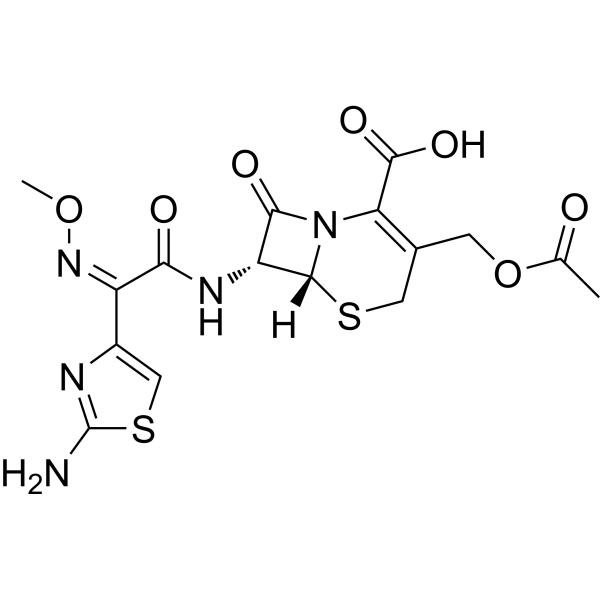 | [10] |
|  | **Ceftaroline fosamil** (Beta-lactam) | 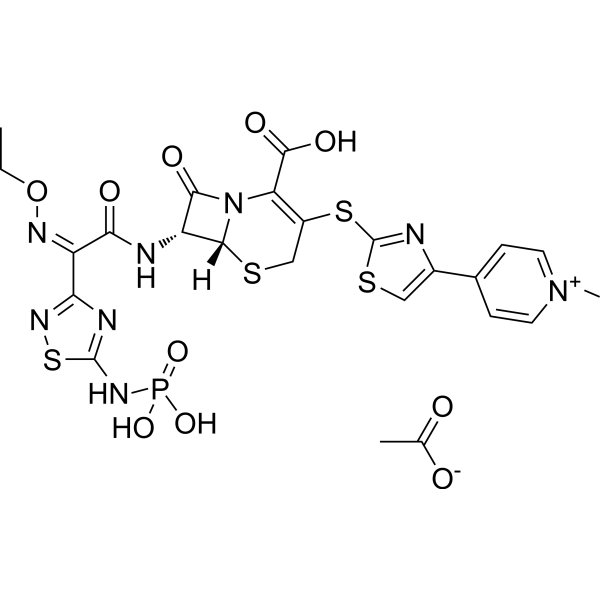 | [11] |
|  | Ertapenem sodium (Beta-lactam) | 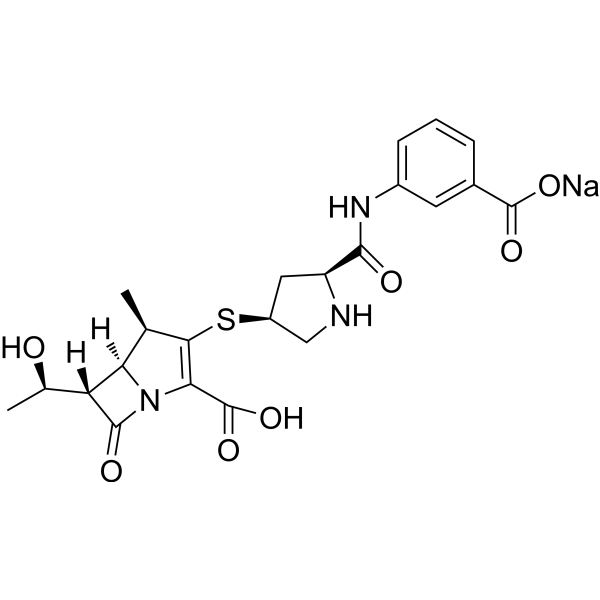 | [12] |
|  | Biapenem (Beta-lactam) | 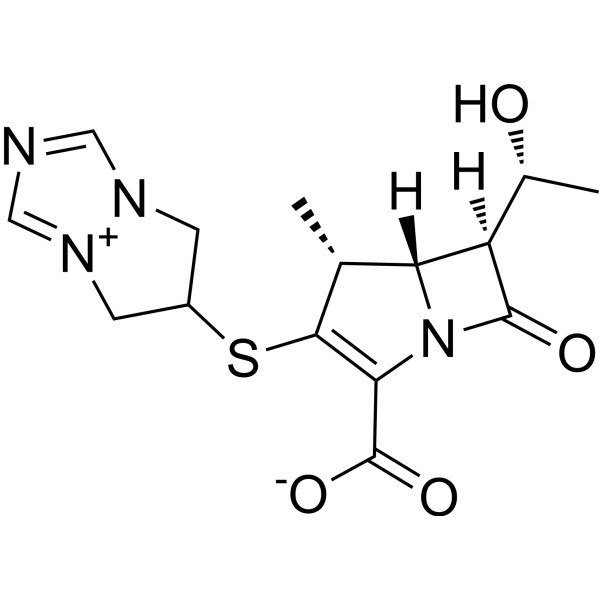 | [13] |
|  | Sultamicillin (Beta-lactam) | 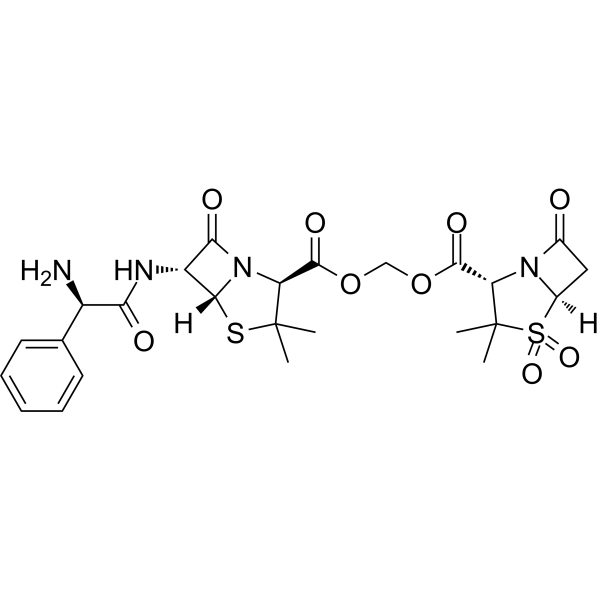 | [14] |
|  | Ampicillin trihydrate (Beta-lactam) | 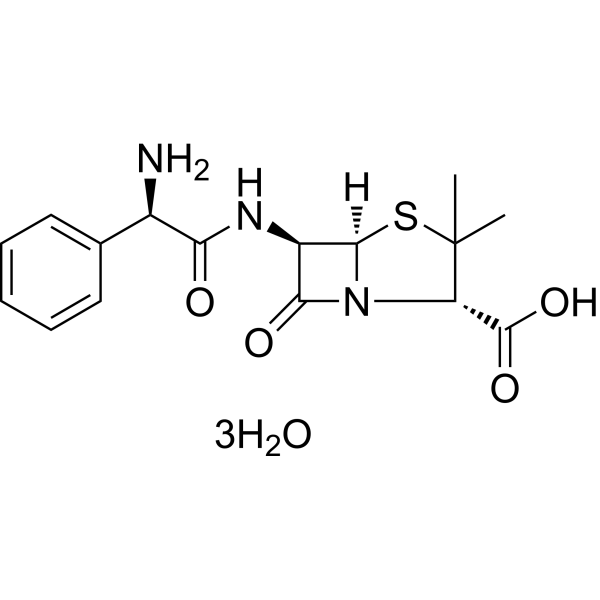 | [15] |
|  | Cefonicid sodium (Beta-lactam) | 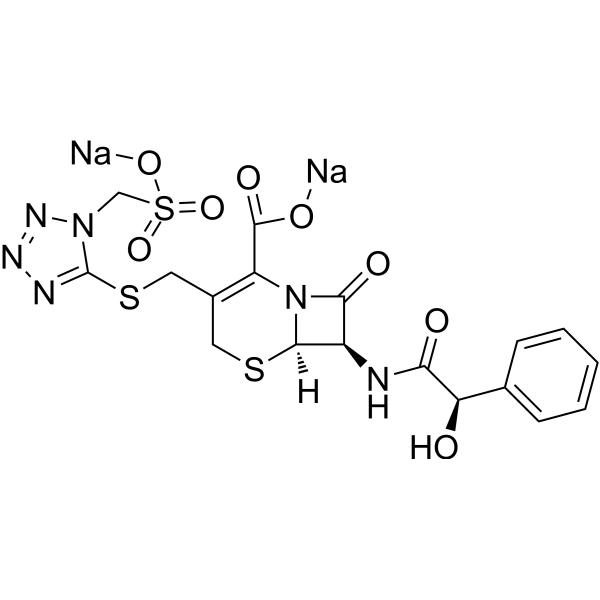 | [16] |
|  | Aspoxicillin (Beta-lactam) | 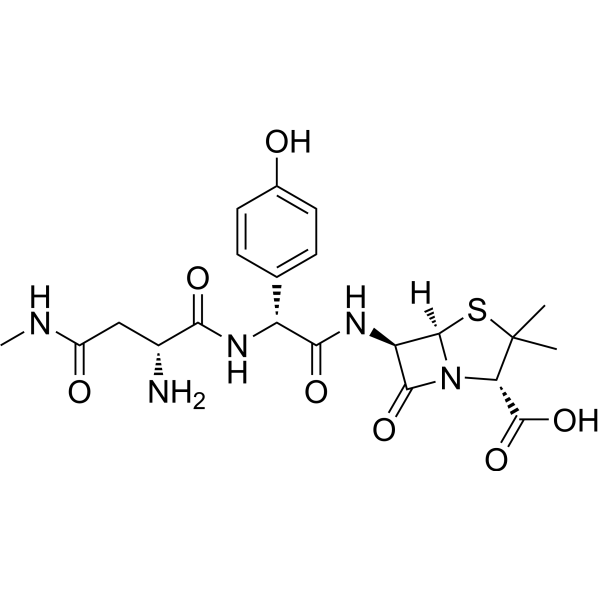 | This study |
|  | Mezlocillin sodium (Beta-lactam) | 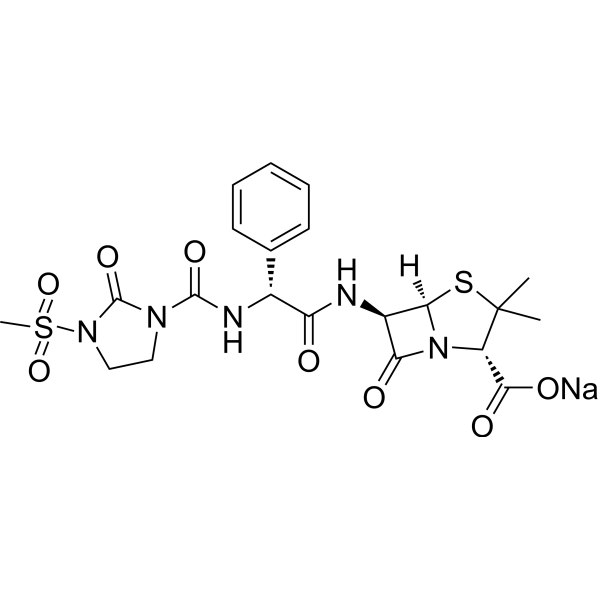 | [7] |
|  | Flomoxef sodium (Beta-lactam) | 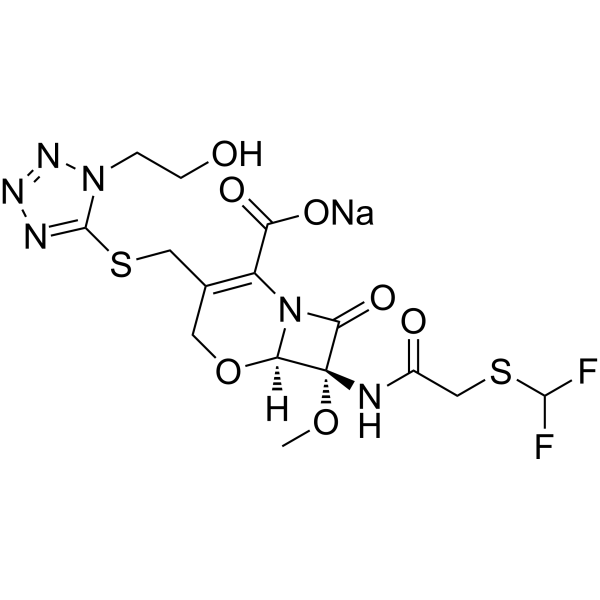 | [17] |
|  | (2S,5R,6R)-Ticarcillin disodium (Beta-lactam) | 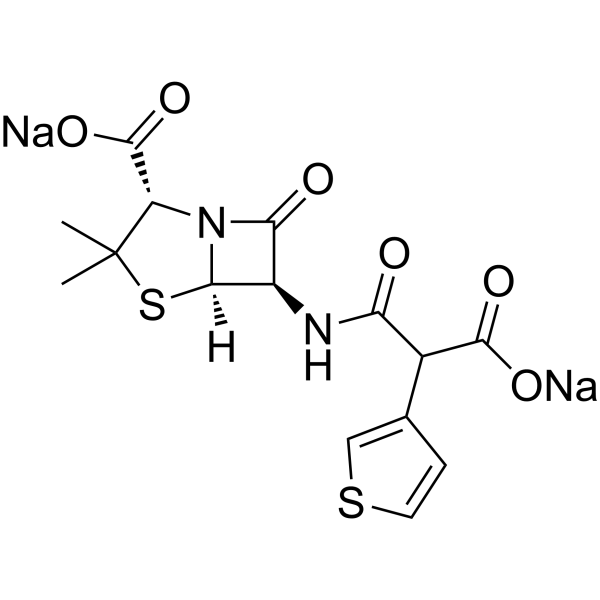 | [18] |
|  | Cefdinir (Beta-lactam) | 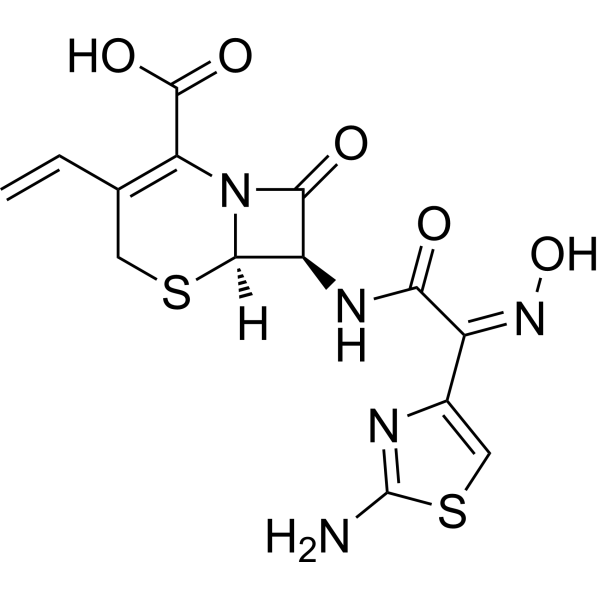 | [19] |
|  | Ceftizoxime(Beta-lactam) | 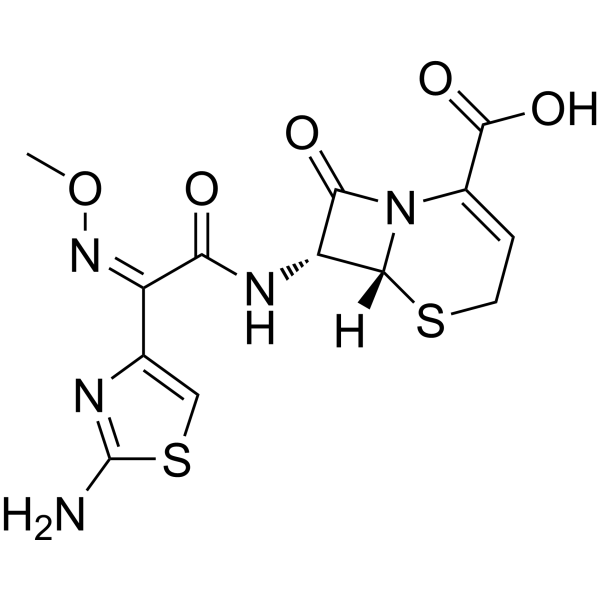 | [6] |
|  | Doripenem monohydrate (Beta-lactam) | 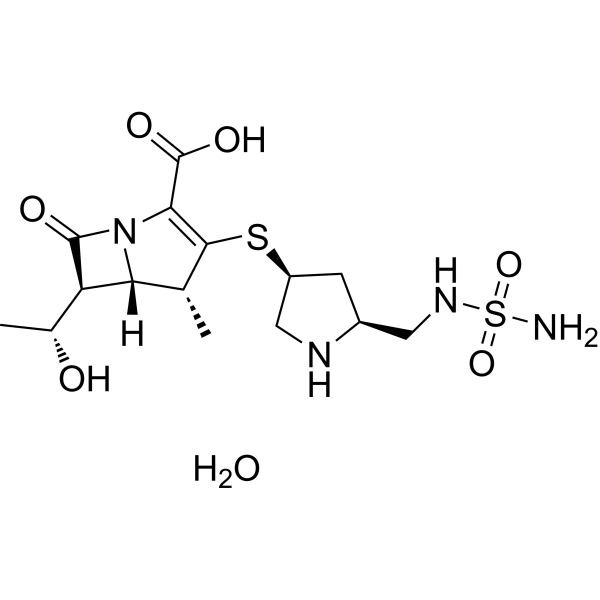 | [20] |
|  | **Ceftazidime pentahydrate** (Beta-lactam) | 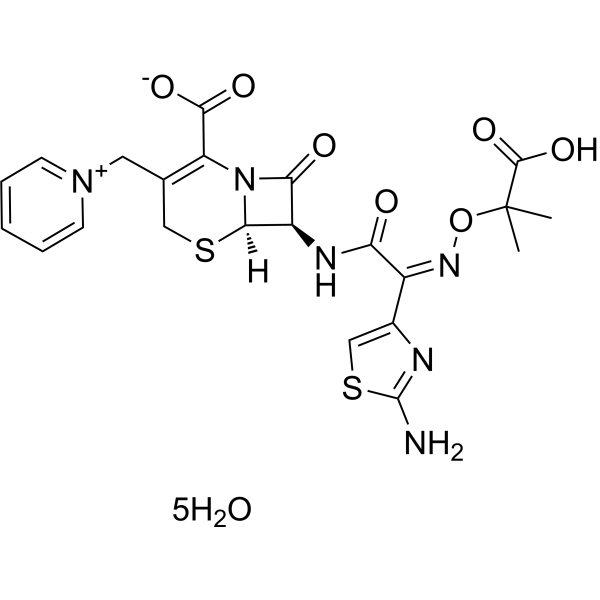 | [21] |
|  | **Tazobactam sodium** (Beta-lactam) | 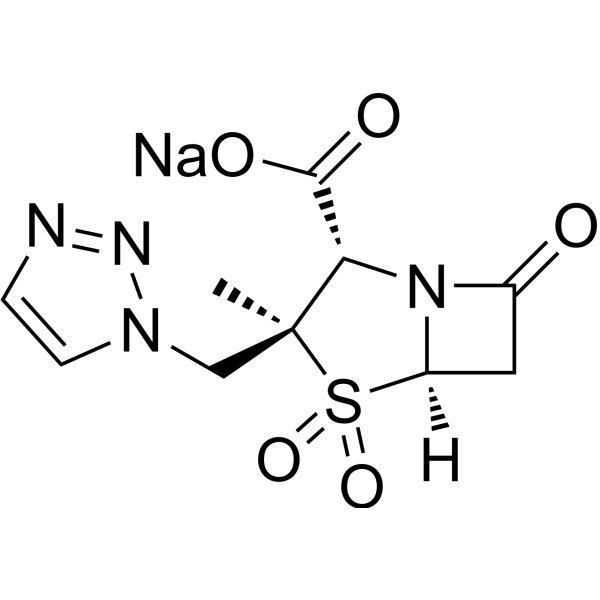 | [22] |
|  | Meropenem trihydrate (Beta-lactam) | 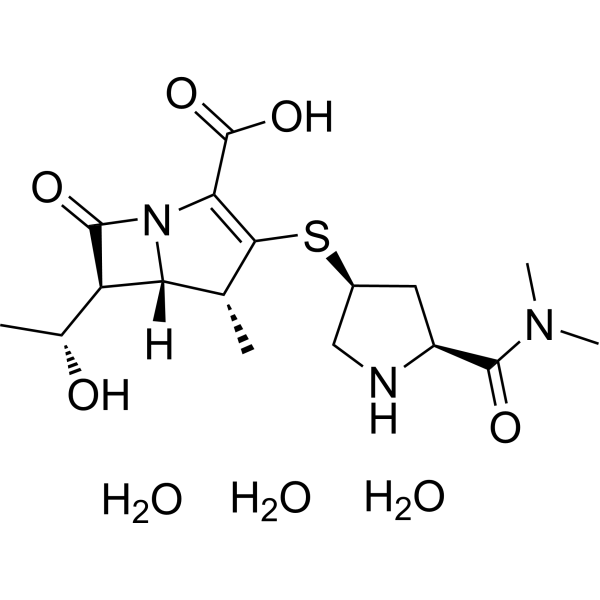 | [23] |
|  | Cefuroxime (Beta-lactam) | 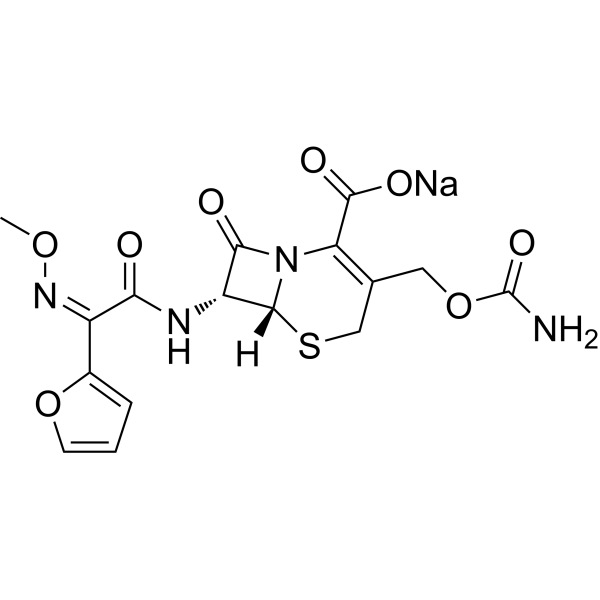 | [24] |
|  | Cefquinome sulfate (Beta-lactam) | 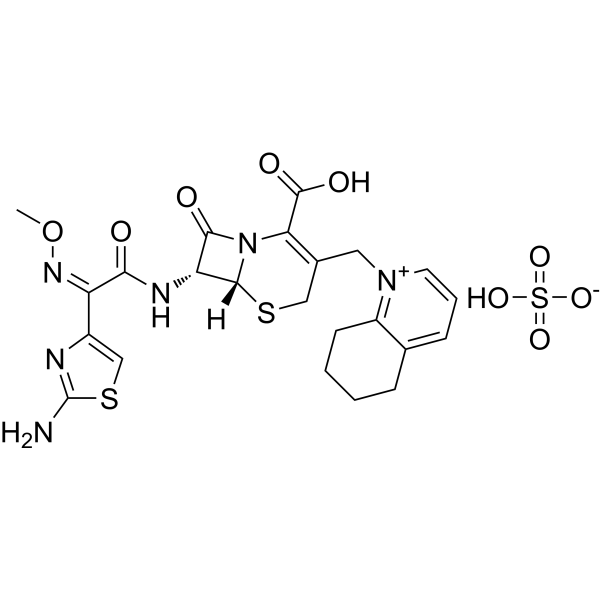 | [25] |
|  | **Cefodizime** (Beta-lactam) | 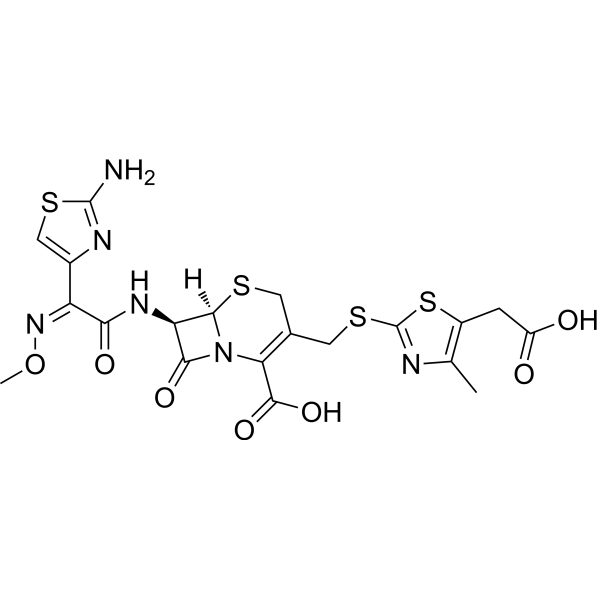 | [21] |
|  | Faropenem daloxate (Beta-lactam) | 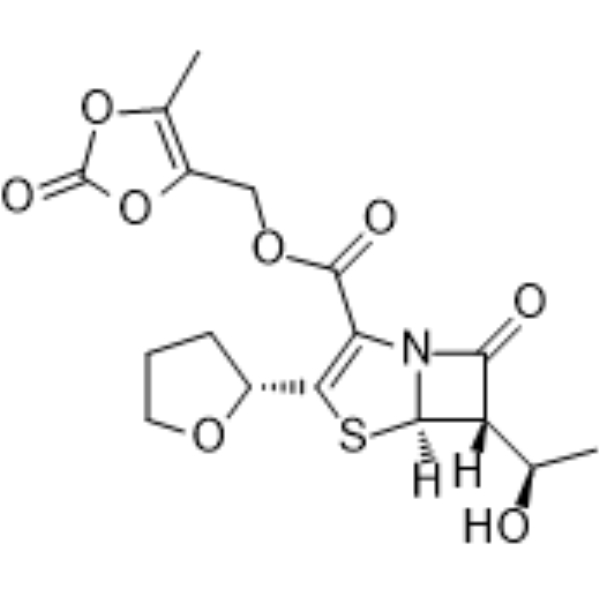 | [26] |
|  | Faropenem sodium (Beta-lactam) | 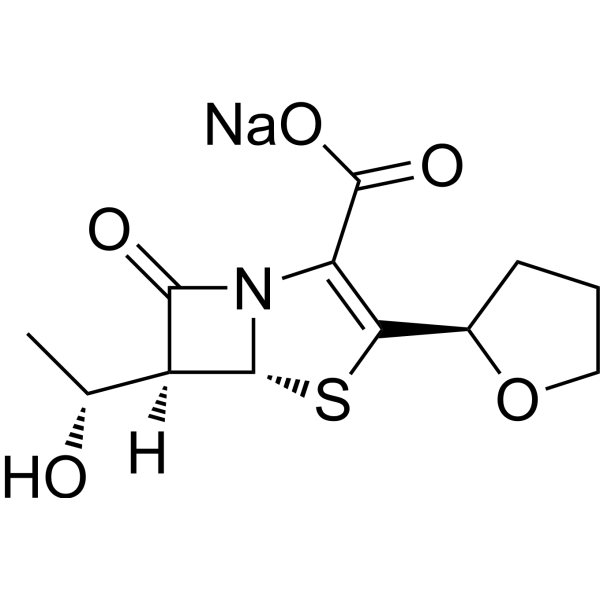 | [26] |
|  | Ceftiofur (Beta-lactam) | 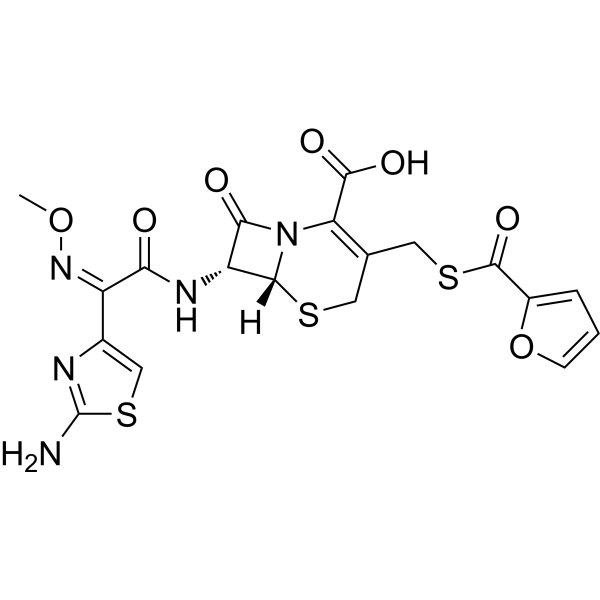 | This study |
|  | Aztreonam (Beta-lactam) | 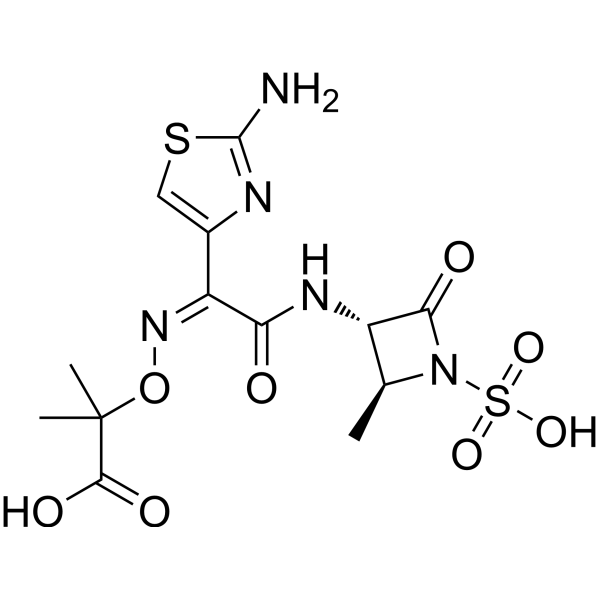 | [27] |
|  | Penicillin G procaine hydrate (Beta-lactam) | 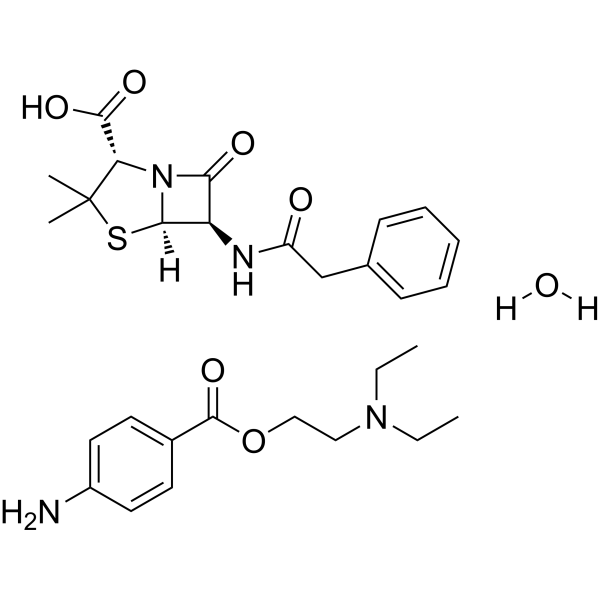 | [28] |
|  | **Penicillin G benzathine tetrahydrate** (Beta-lactam) | 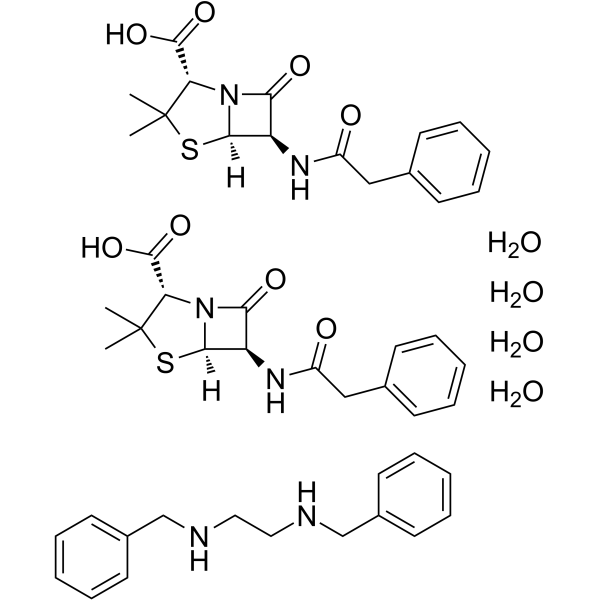 | [28] |
|  | Azlocillin sodium salt (Beta-lactam) | 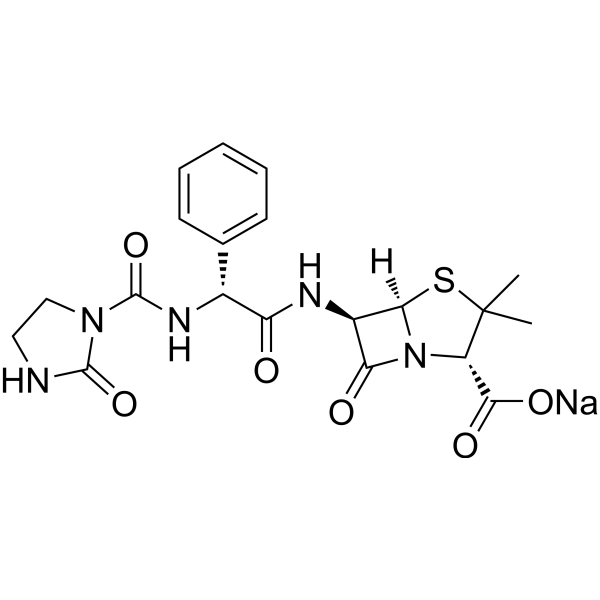 | [7] |
|  | Cefmenoxime (Beta-lactam) | 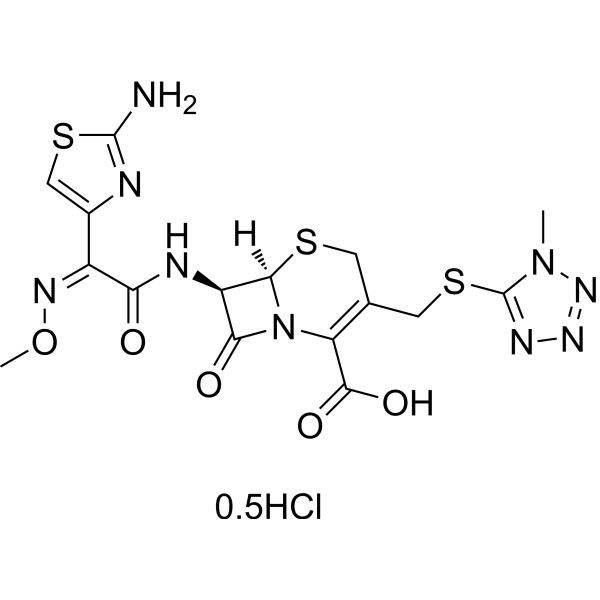 | [24] |
|  | Cephalothin (Beta-lactam) | 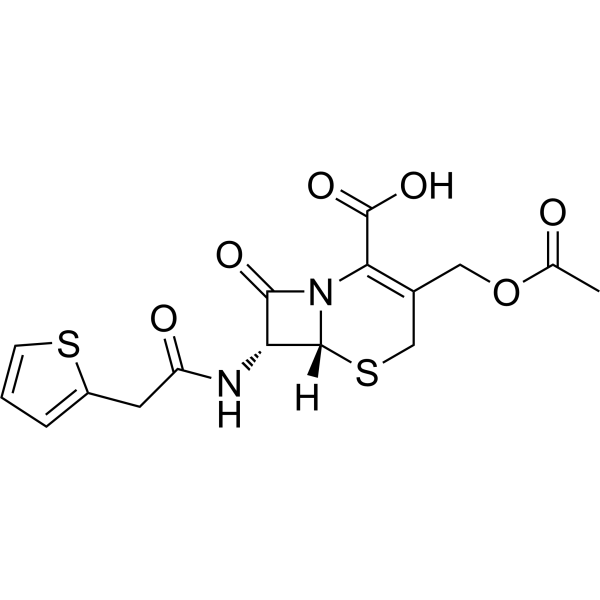 | [7] |
|  | Piperacillin (Beta-lactam) | 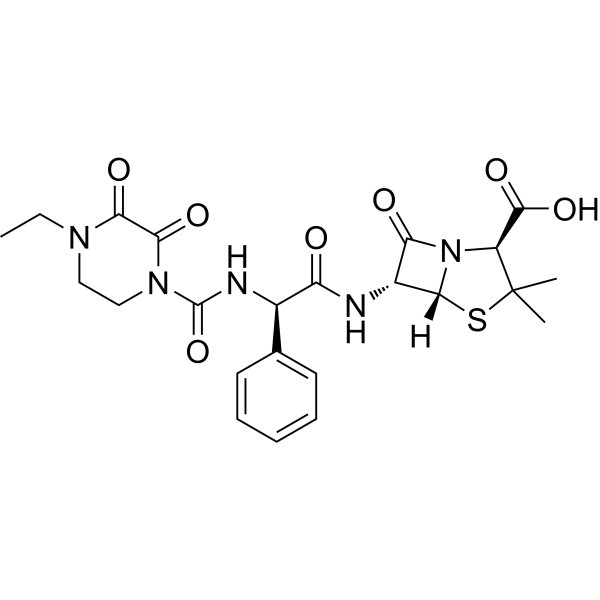 | [7] |
|  | Cefepime Dihydrochloride Monohydrate (Beta-lactam) | 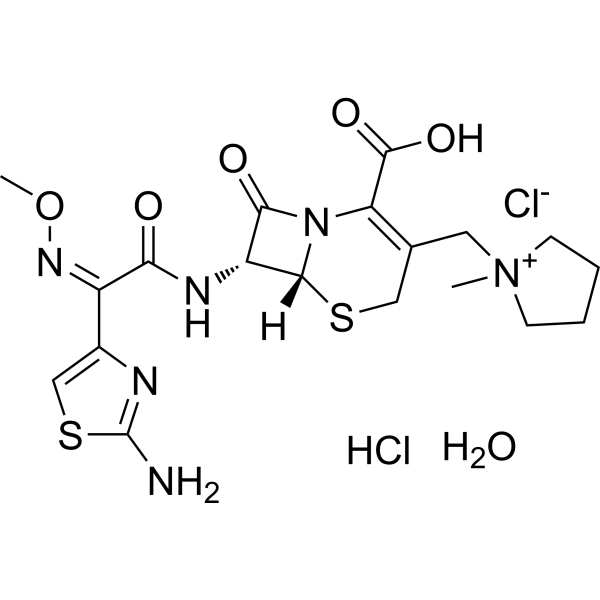 | [25] |
|  | Ceftibuten dihydrate (Beta-lactam) | 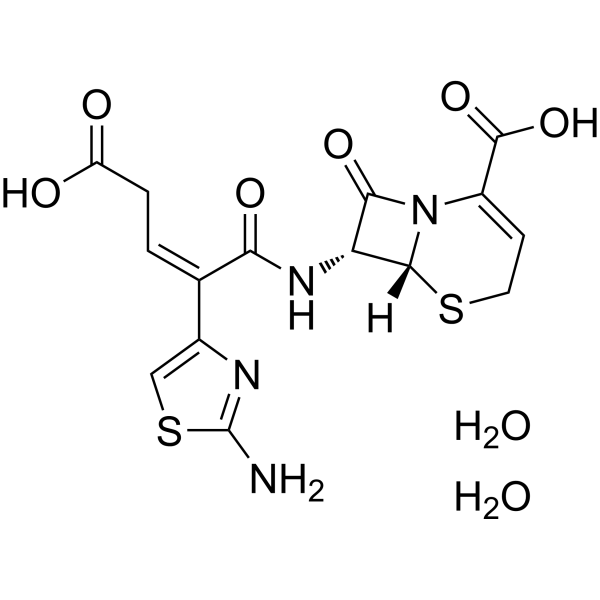 | [29] |
|  | Cefditoren sodium (Beta-lactam) | 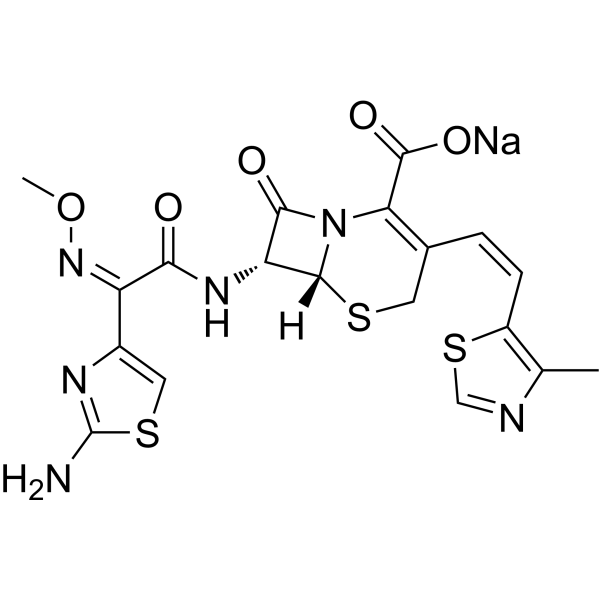 | [30] |
|  | Cefixime (Beta-lactam) | 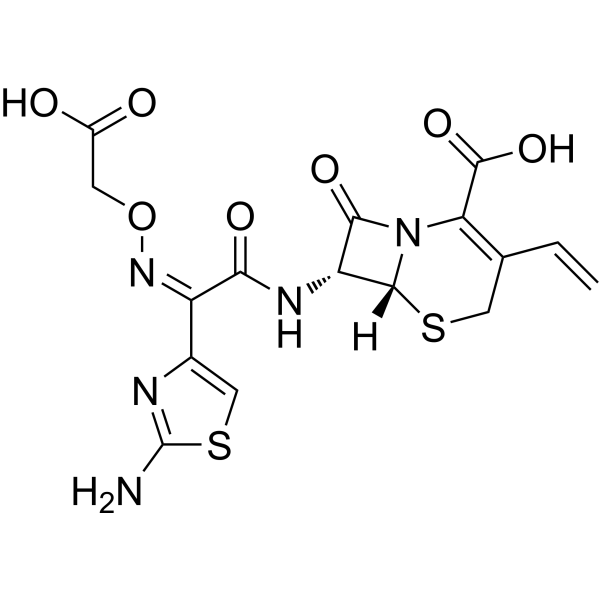 | [31] |
|  | Cefozopran (Beta-lactam) | 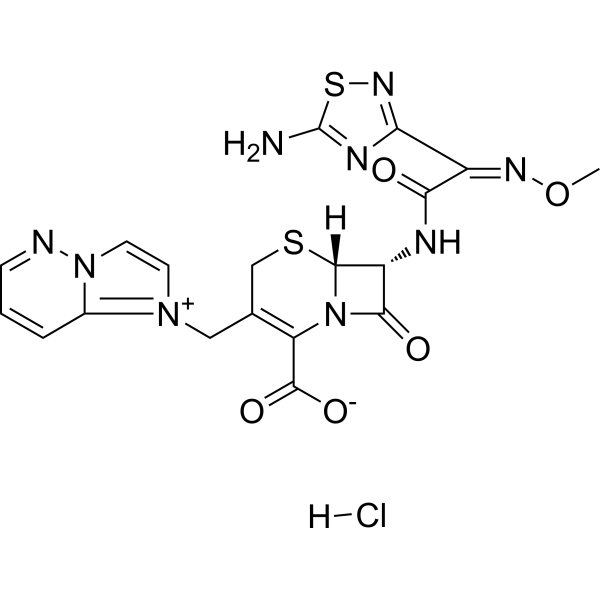 | [32] |
|  | Cefpodoxime (Beta-lactam) | 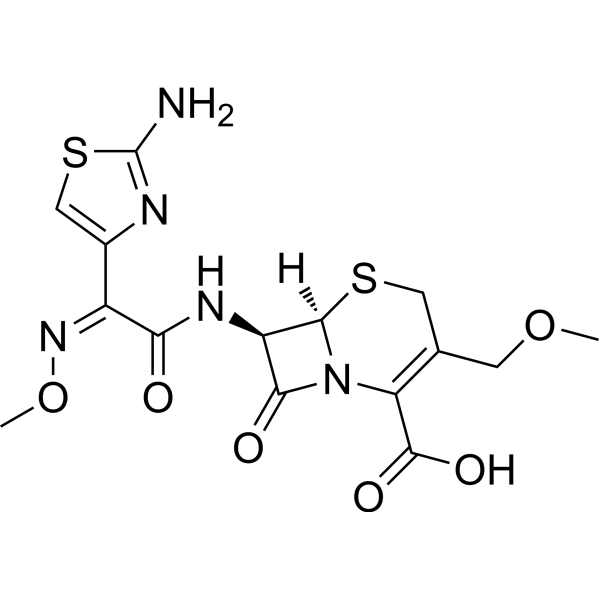 | [19] |
|  | Cefcapene pivoxil hydrochloride (Beta-lactam) | 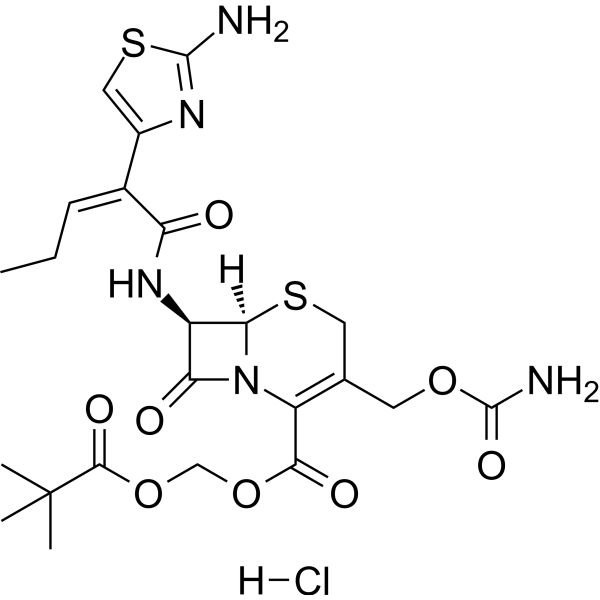 | [33] |
|  | Erythromycin  (Macrolide) | 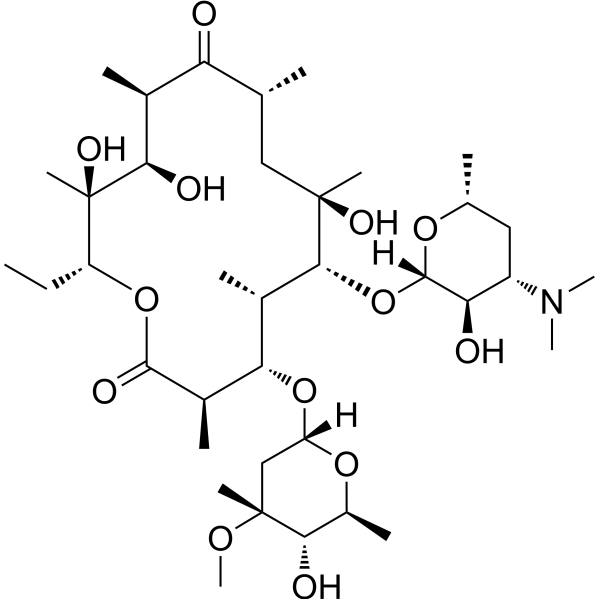 | [34] |
|  | Azathramycin  (Macrolide) | 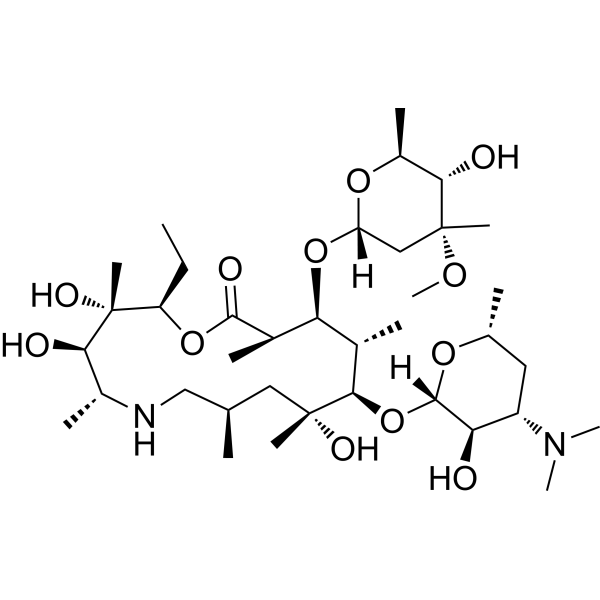 | This study |
|  | Azithromycin  (Macrolide) | 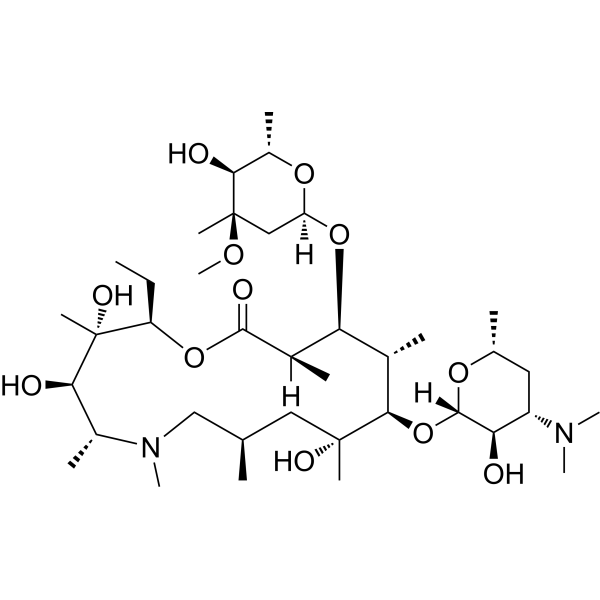 | [34] |
|  | Midecamycin  (Macrolide) | 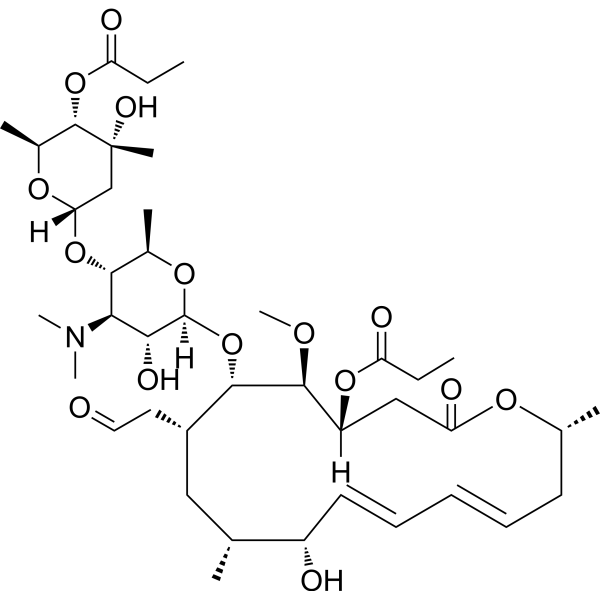 | [35] |
|  | Leucomycin  (Macrolide) | 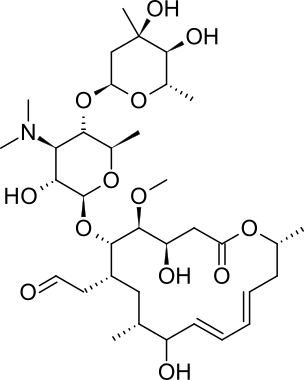 | [36] |
|  | Clarithromycin  (Macrolide) | 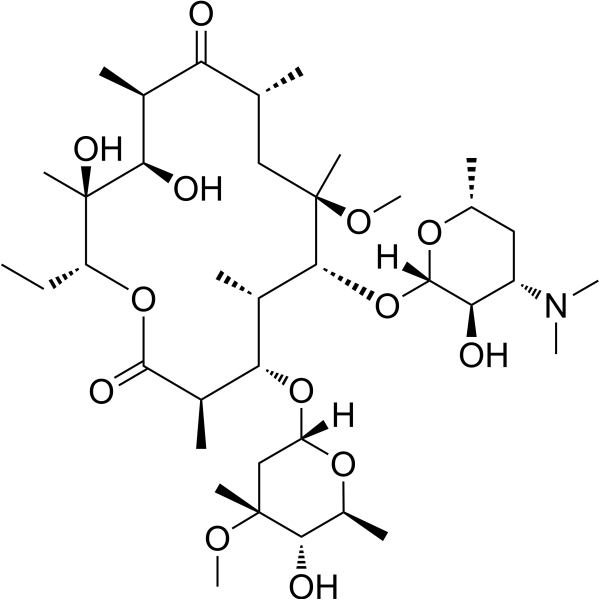 | [34] |
|  | Solithromycin  (Macrolide) | 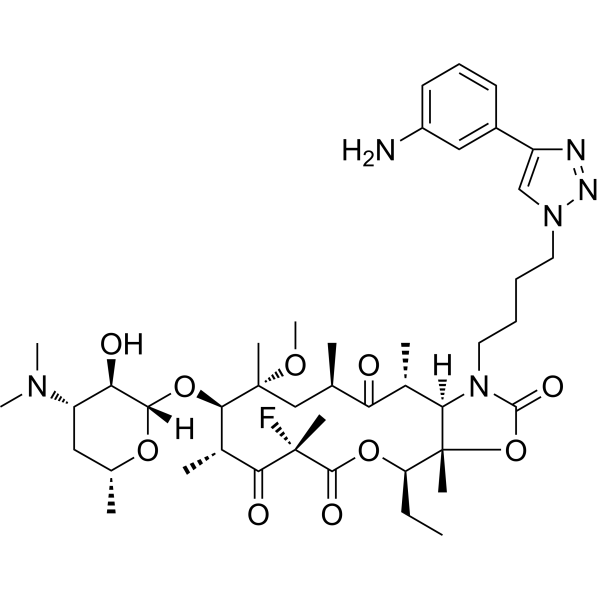 | [37] |
|  | Gamithromycin  (Macrolide) | 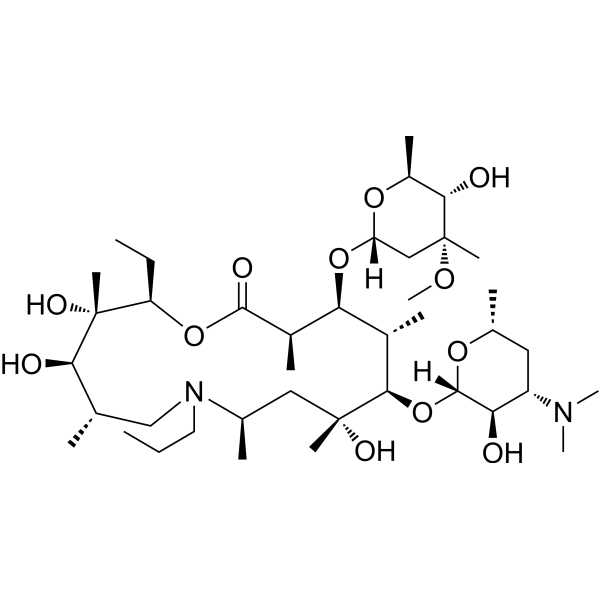 | [9] |
|  | Josamycin  (Macrolide) | 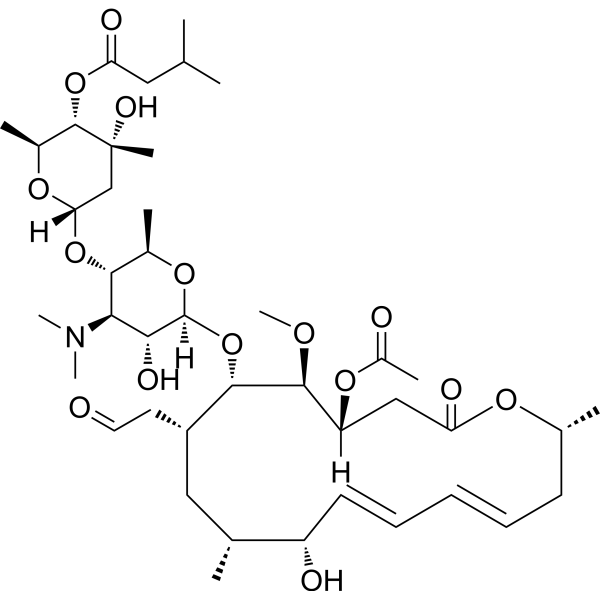 | [38] |
|  | Telithromycin  (Macrolide) | 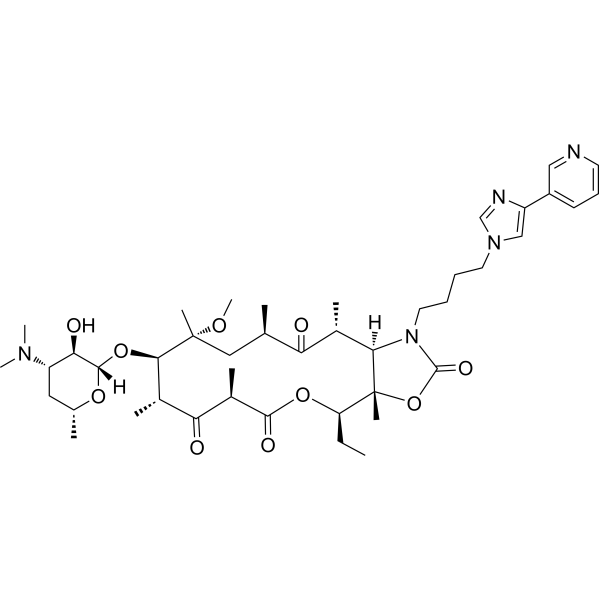 | [39] |
|  | Pristinamycin  (Macrolide) | 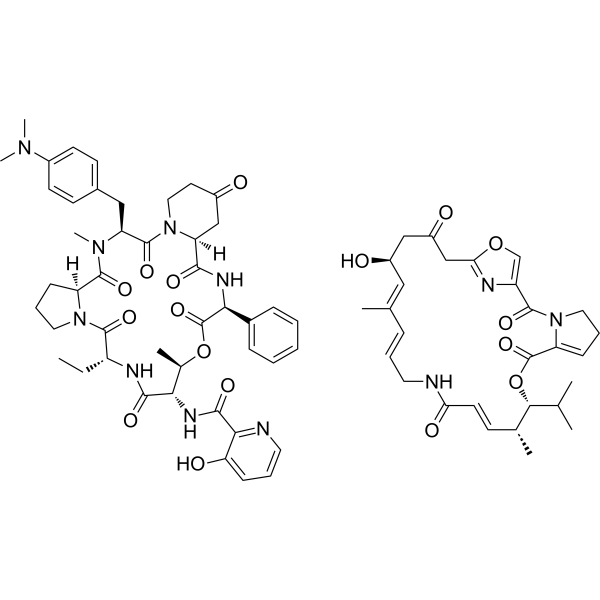 | [40] |
|  | Virginiamycin M1  (Macrolide) | 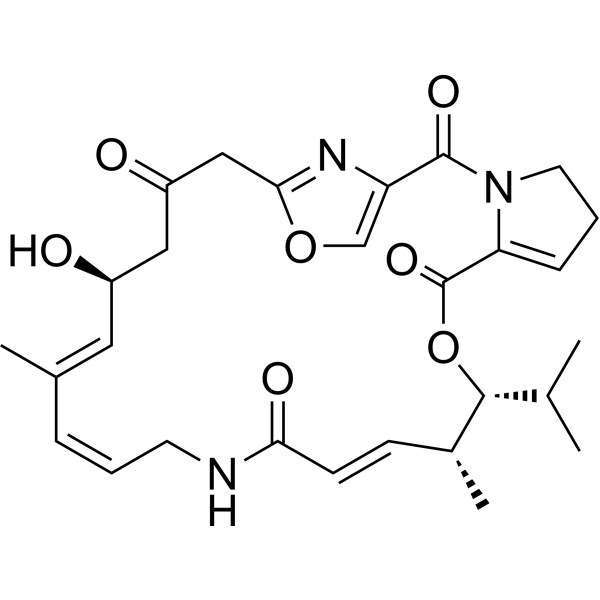 | This study |
|  | Retapamulin  (Macrolide) | 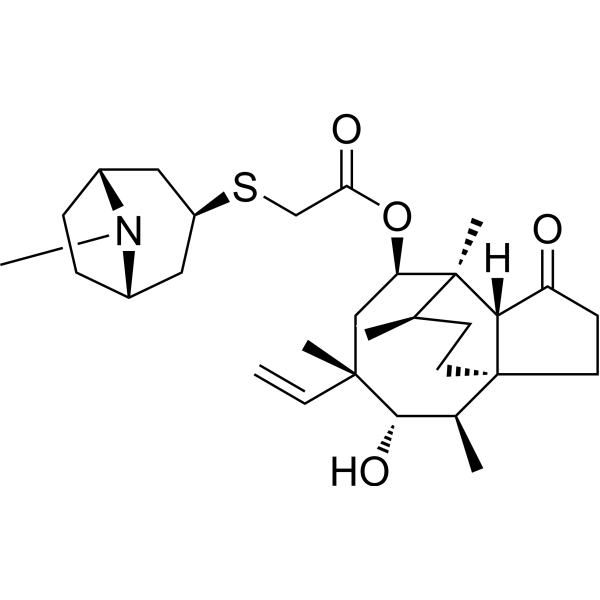 | [9] |
|  | Tiamulin fumarate  (Macrolide) | 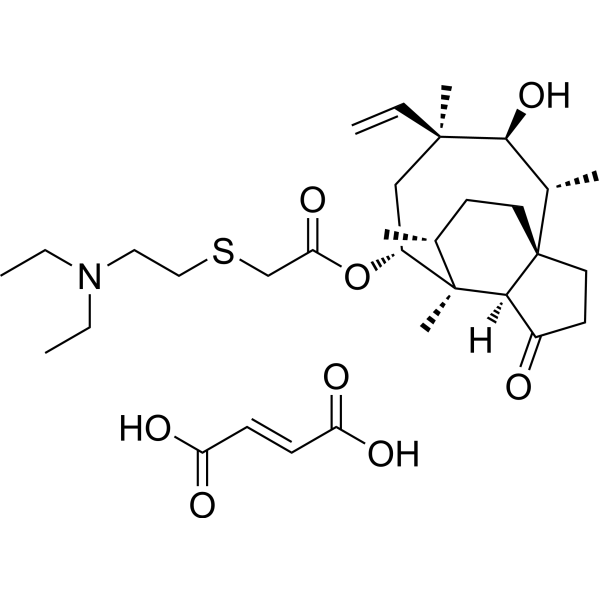 | [9] |
|  | Valnemulin (hydrochloride) | 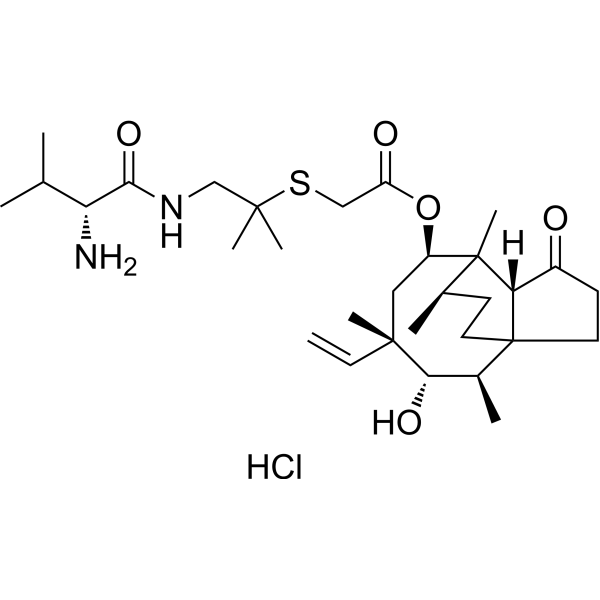 | [9] |
|  | Florfenicol  (Phenicol) | 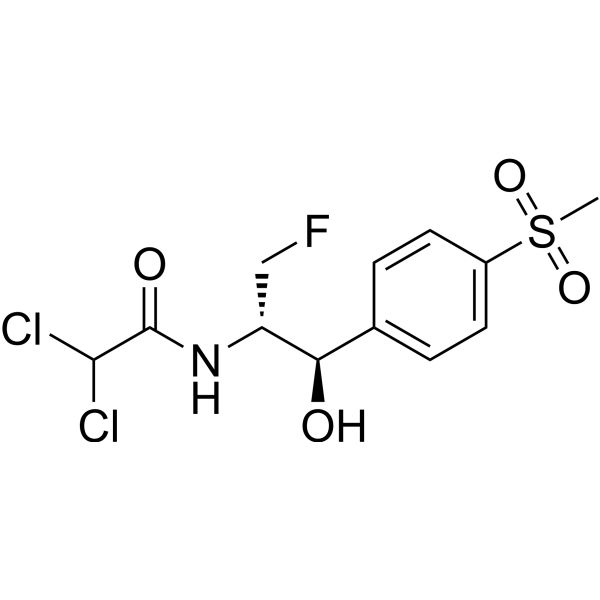 | [41] |
|  | Thiamphenicol  (Phenicol) | 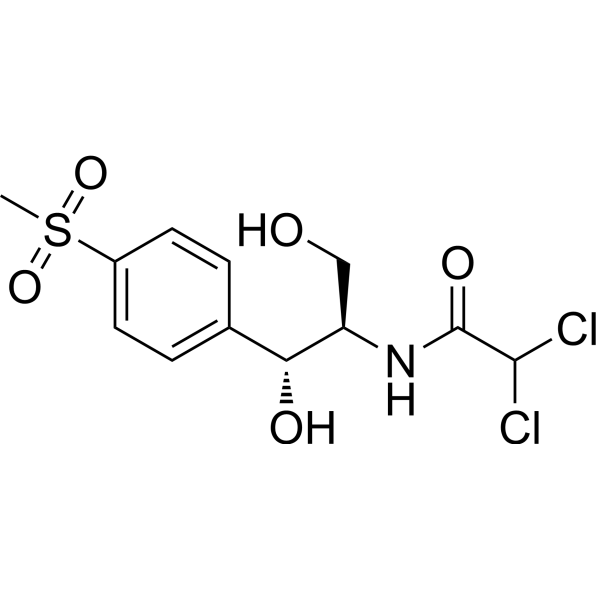 | [42] |
|  | Rifampicin  (Rifamycin) | 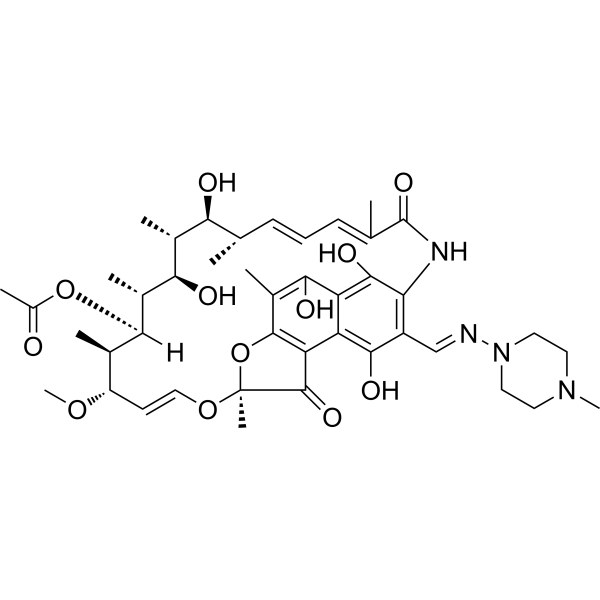 | [43] |
|  | Rifamycin S  (Rifamycin) | 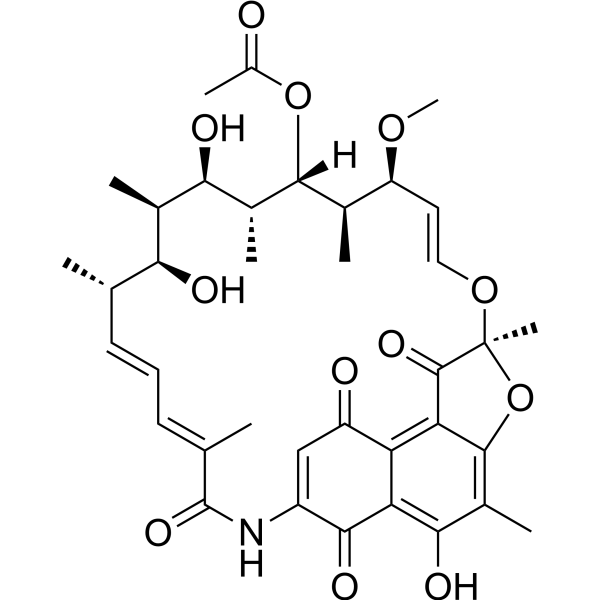 | [44] |
|  | Rifapentine  (Rifamycin) | 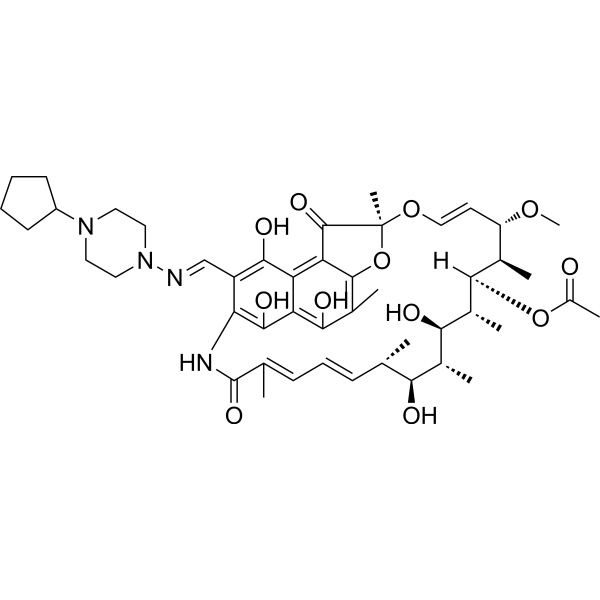 | [45] |
|  | Rifalazil  (Rifamycin) | 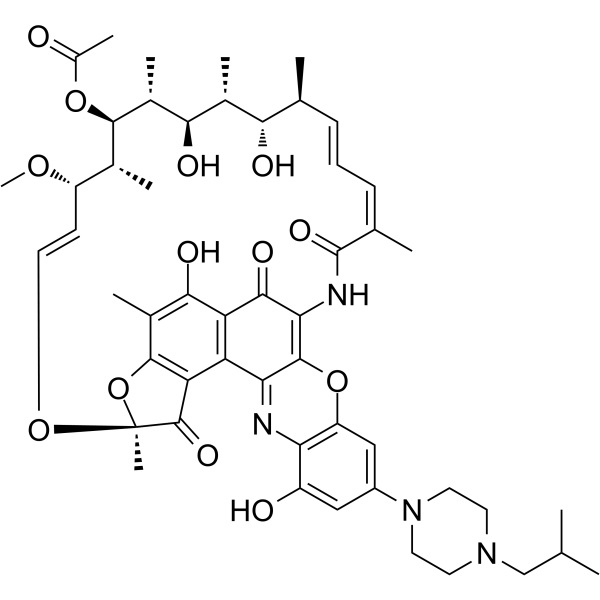 | [45] |
|  | Rifabutin  (Rifamycin) | 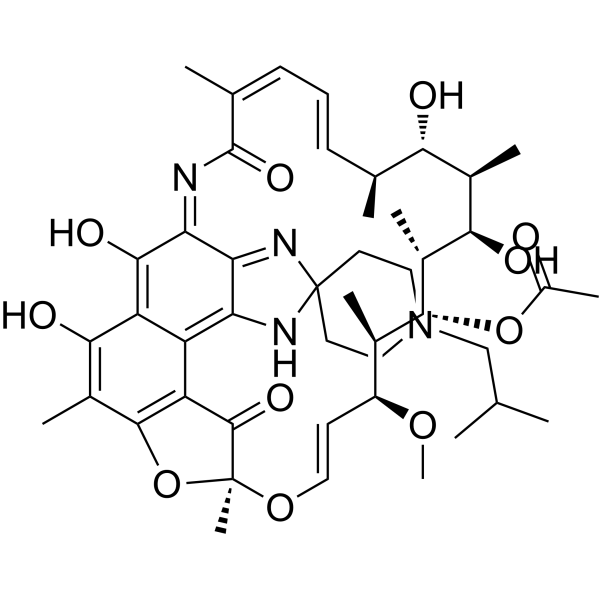 | [43] |
|  | Demeclocycline hydrochloride  (Tetracycline) | 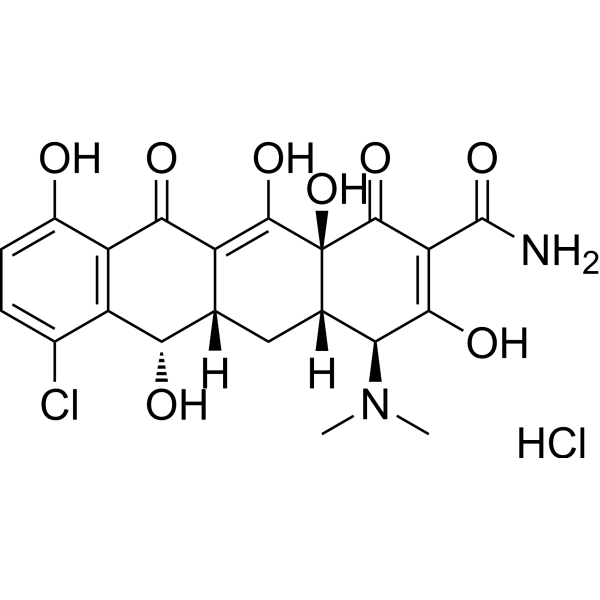 | [46] |
|  | Chlortetracycline hydrochloride  (Tetracycline) | 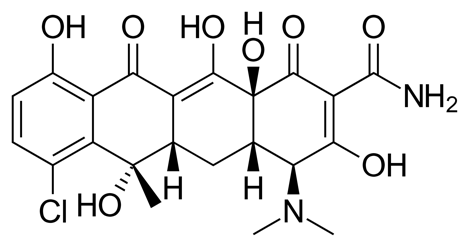 | [46] |
|  | Oxytetracycline  (Tetracycline) | 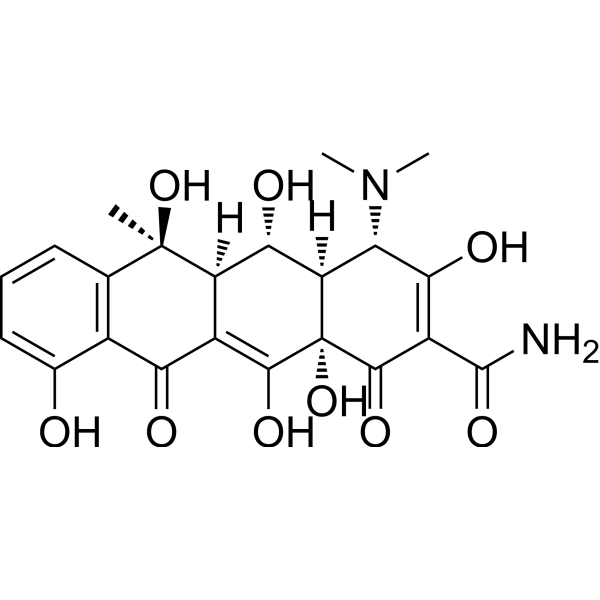 | [46] |
|  | Minocycline hydrochloride  (Tetracycline) | 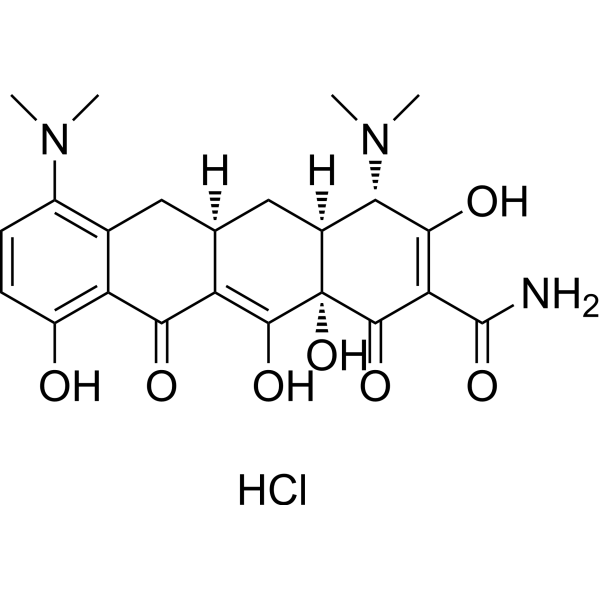 | [46] |
|  | Doxycycline  (Tetracycline) | 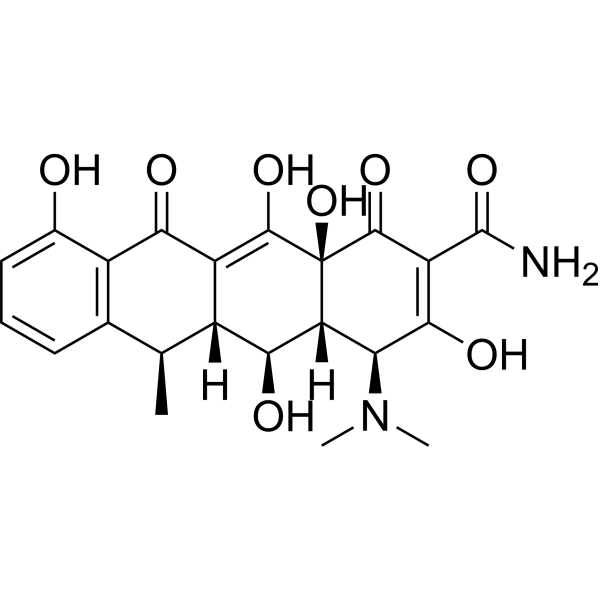 | [46] |
|  | Tetracycline hydrochloride  (Tetracycline) | 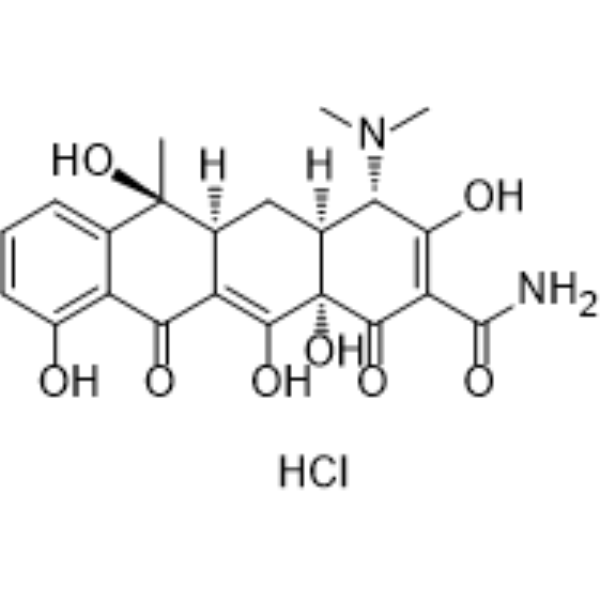 | [46] |
|  | Tigecycline tetramesylate  (Tetracycline) | 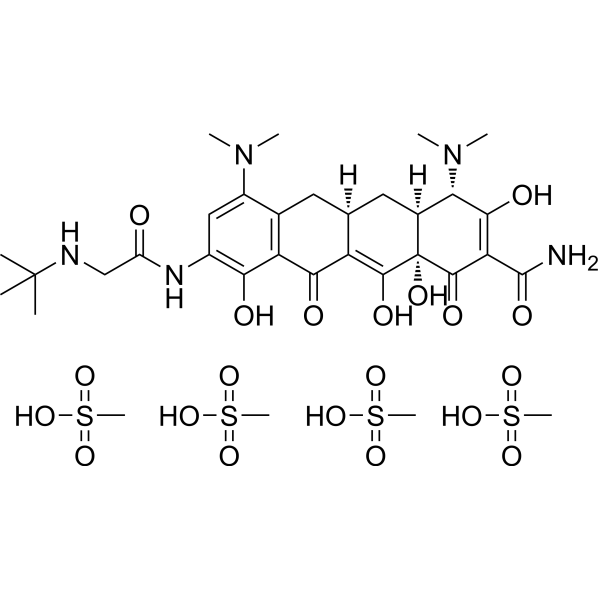 | [47] |
|  | Methacycline hydrochloride  (Tetracycline) | 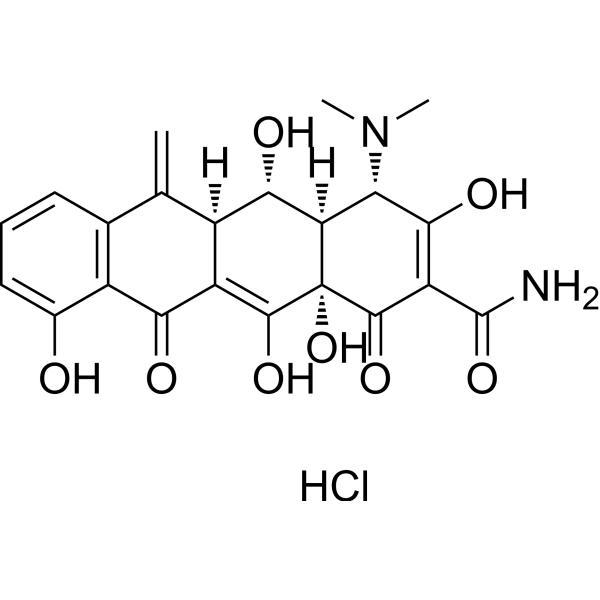 | [46] |
|  | Tosufloxacin  (Quinolone) | 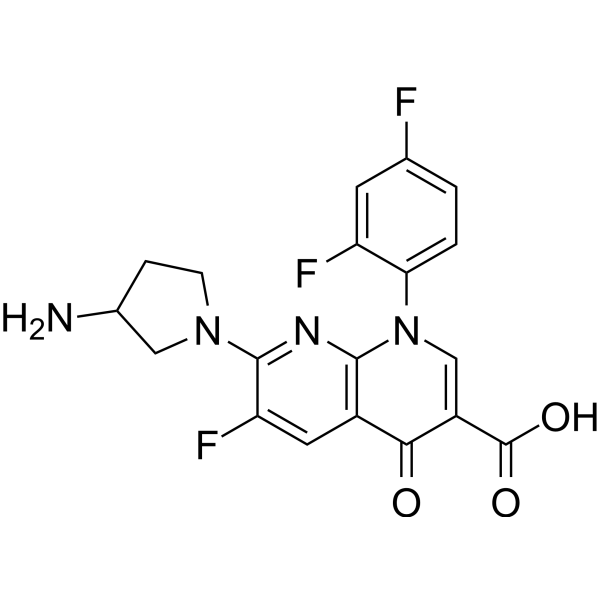 | [48] |
|  | Sparfloxacin  (Quinolone) | 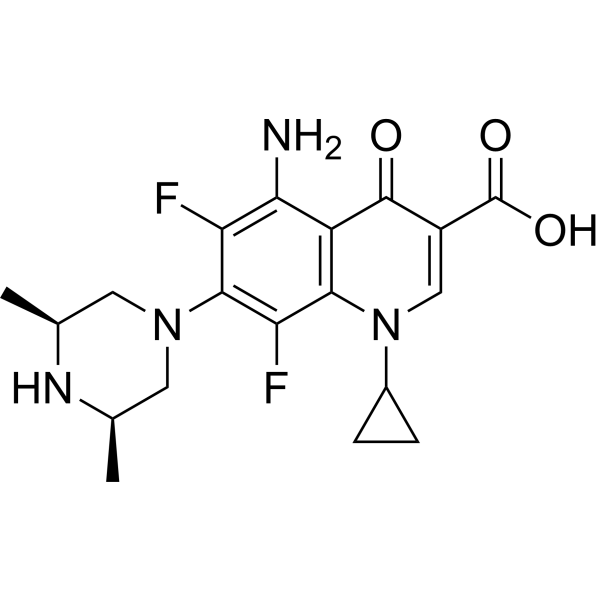 | [49] |
|  | Clinafloxacin  (Quinolone) | 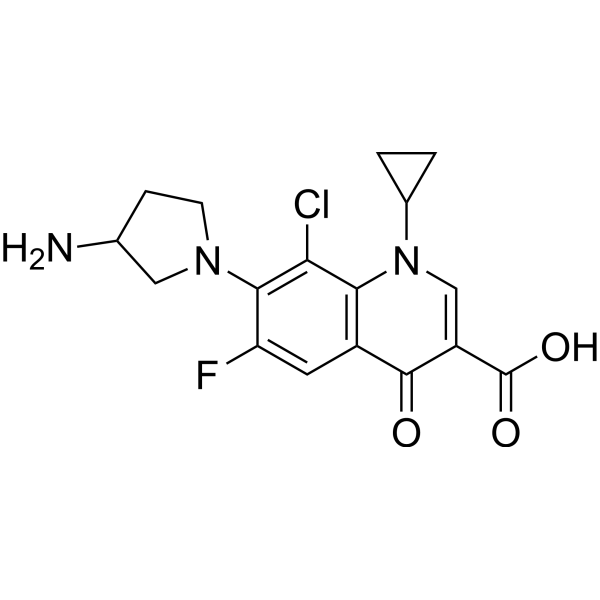 | [50] |
|  | Ciprofloxacin  (Quinolone) | 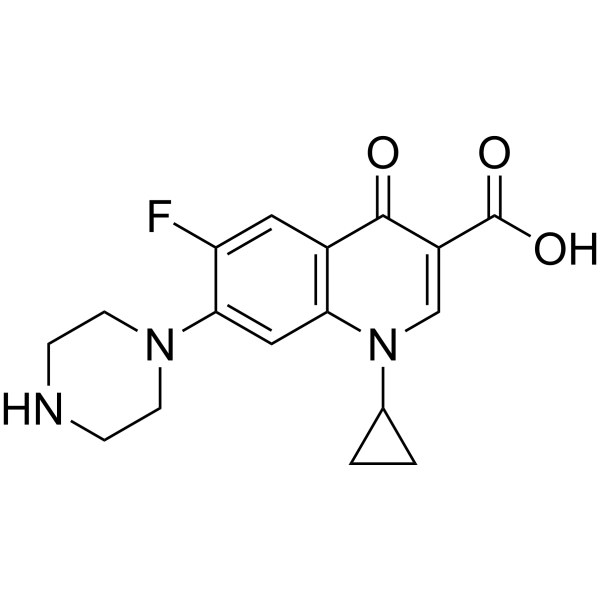 | [50] |
|  | Marbofloxacin  (Quinolone) | 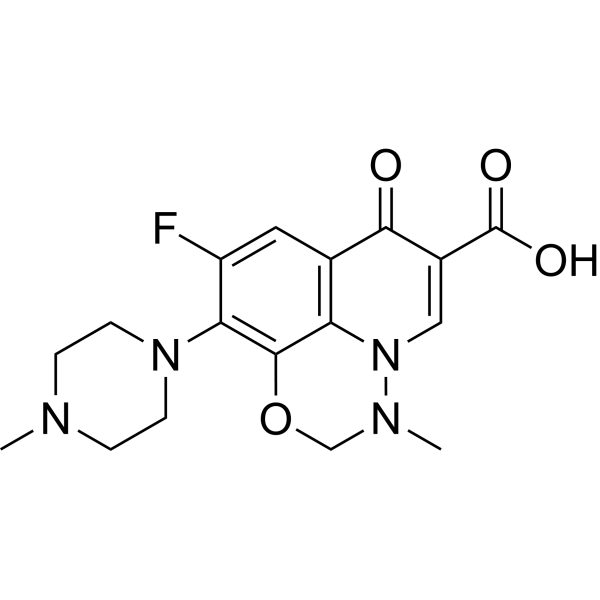 | This study |
|  | Rufloxacin hydrochloride  (Quinolone) | 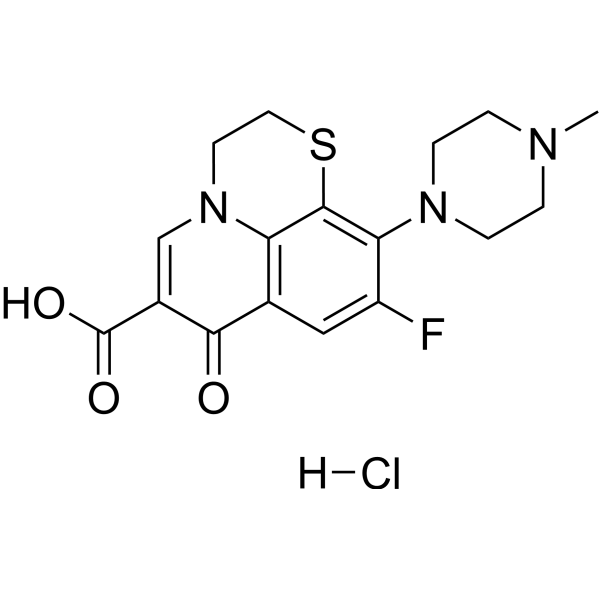 | [51] |
|  | Sitafloxacin hydrate  (Quinolone) | 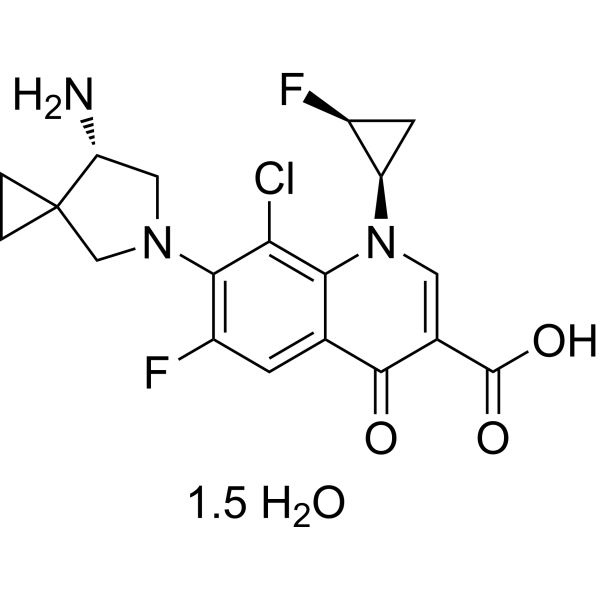 | [52] |
|  | BAY-Y 3118  (Quinolone) | 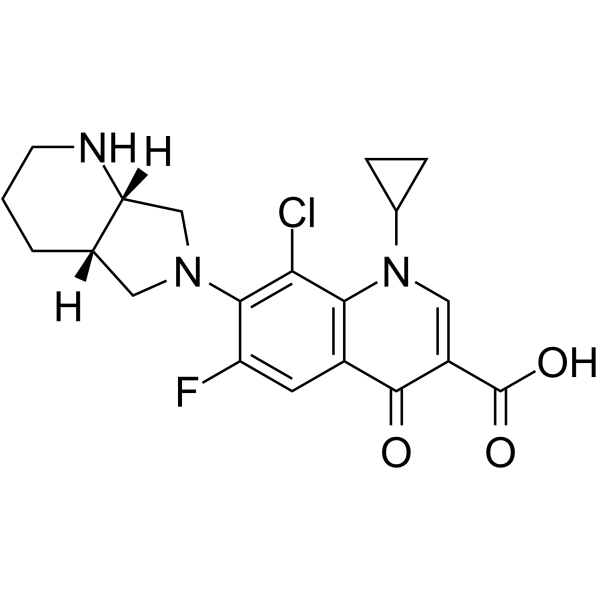 | [50] |
|  | Prulifloxacin  (Quinolone) | 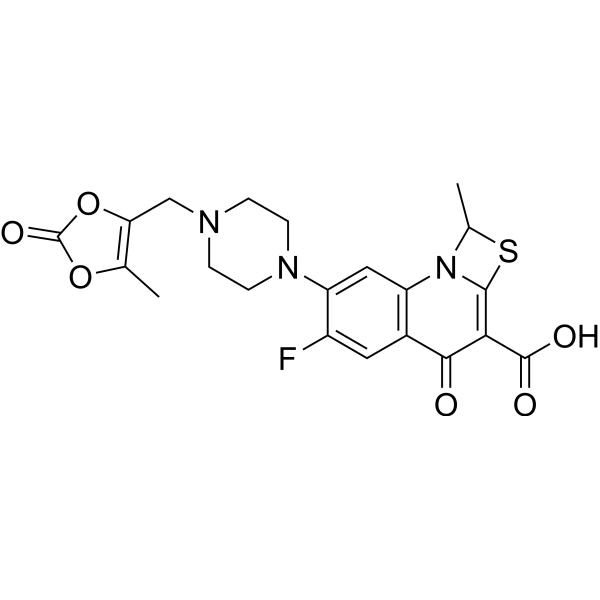 | [53] |
|  | Zabofloxacin hydrochloride  (Quinolone) | 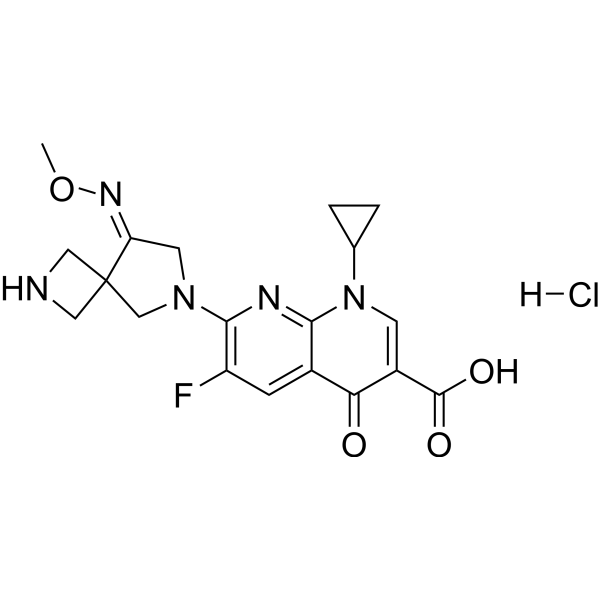 | [54] |
|  | Trovafloxacin  (Quinolone) | 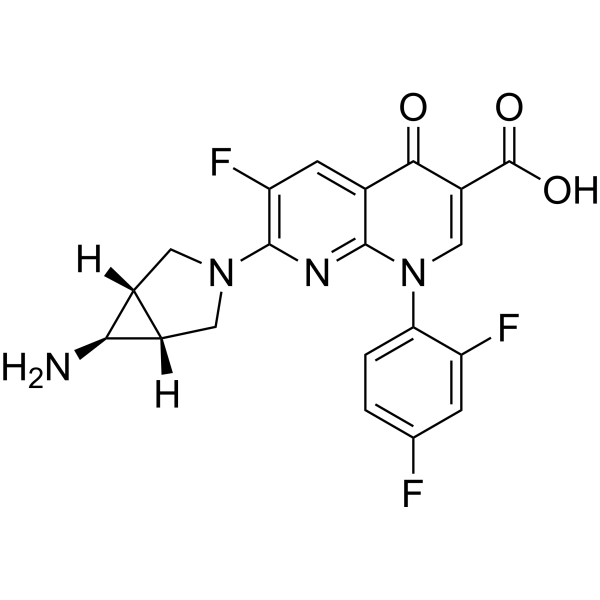 | [55] |
|  | Orbifloxacin  (Quinolone) | 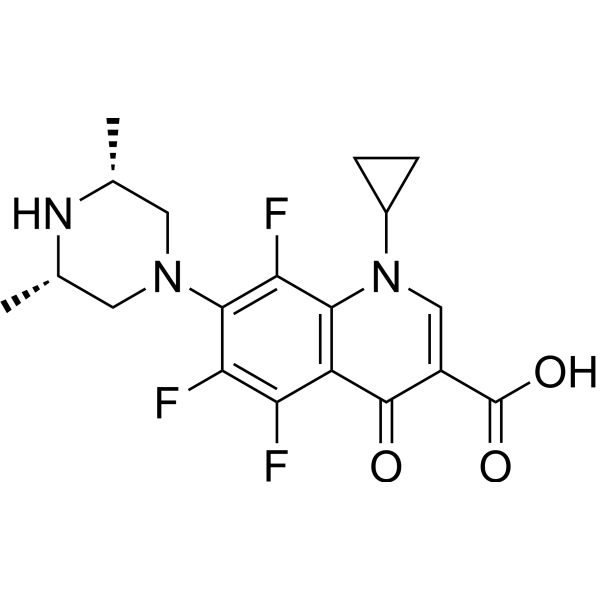 | This study |
|  | Rosoxacin  (Quinolone) | 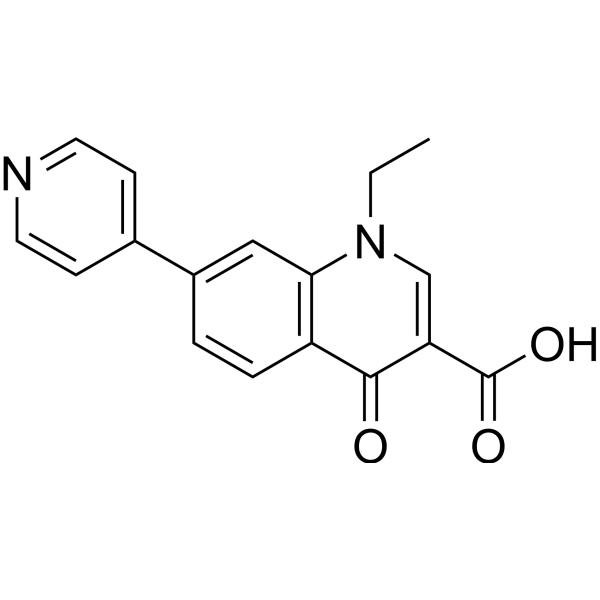 | [56] |
|  | Levonadifloxacin  (Quinolone) | 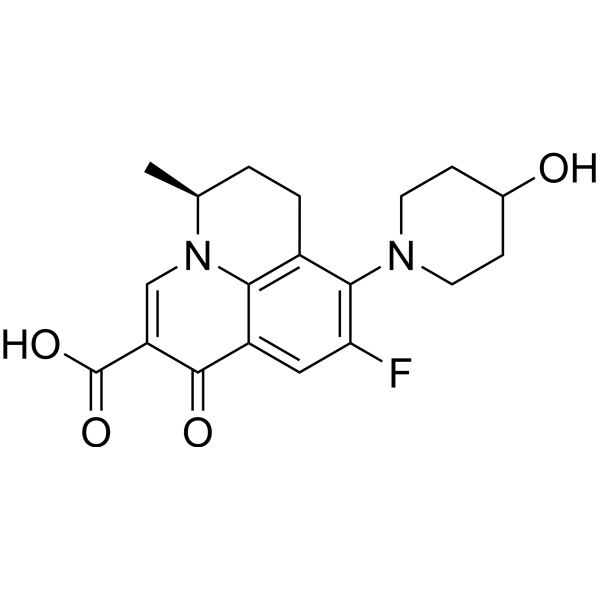 | This study |
|  | Nadifloxacin  (Quinolone) | 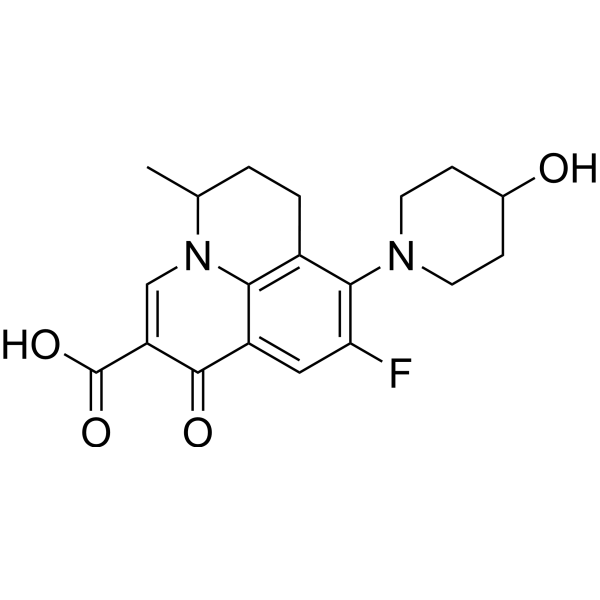 | [41] |
|  | Lomefloxacin hydrochloride  (Quinolone) | 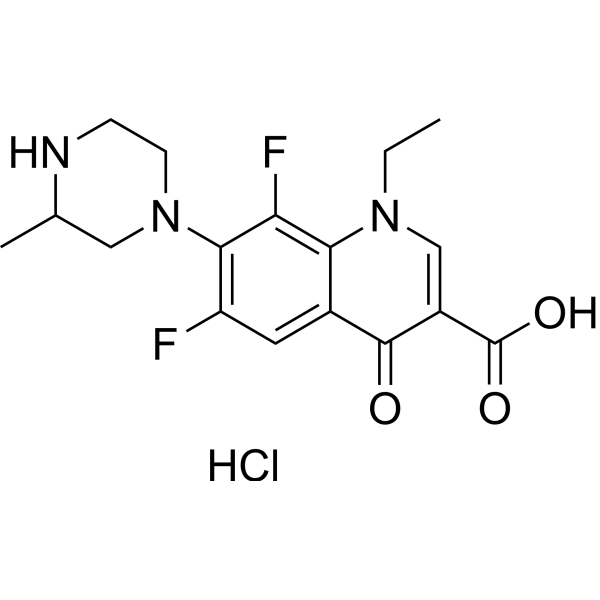 | [49] |
|  | Gemifloxacin mesylate  (Quinolone) | 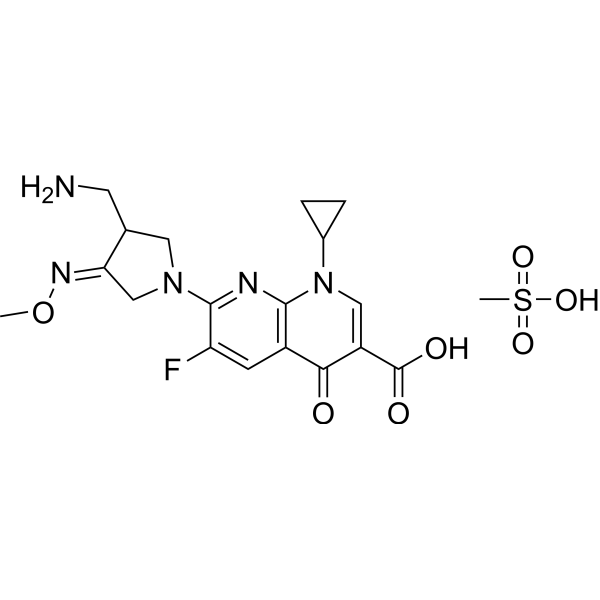 | [57] |
|  | Levofloxacin  (Quinolone) | 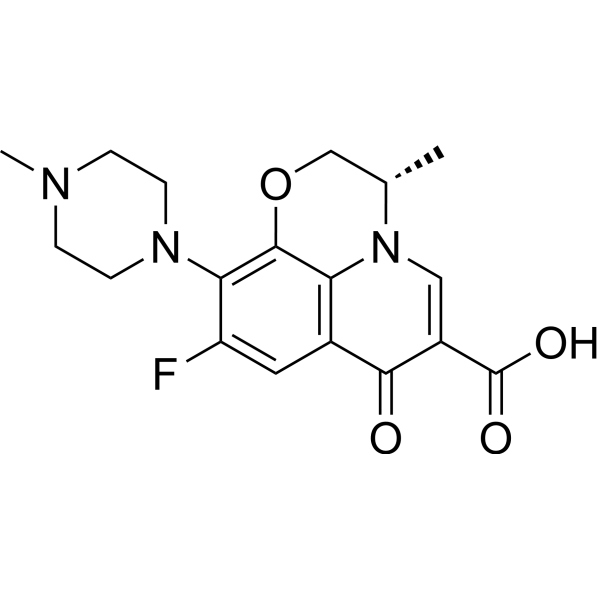 | [57] |
|  | Enoxacin hydrate  (Quinolone) | 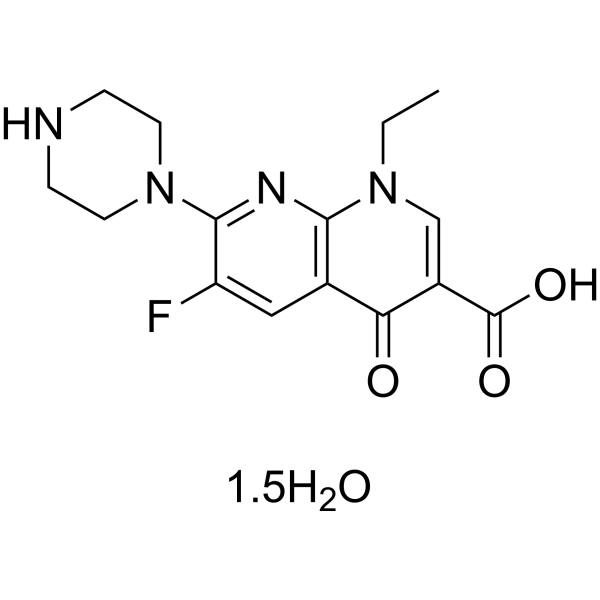 | [58] |
|  | Enrofloxacin monohydrochloride  (Quinolone) | 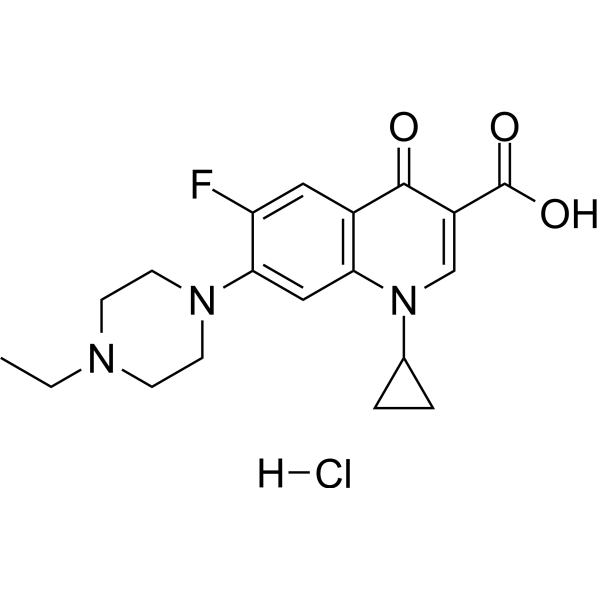 | [59] |
|  | Gatifloxacin  (Quinolone) | 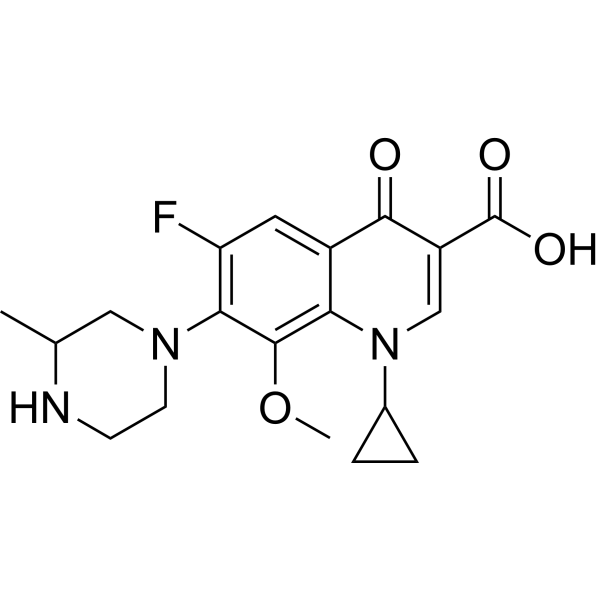 | [60] |
|  | Difloxacin hydrochloride  (Quinolone) | 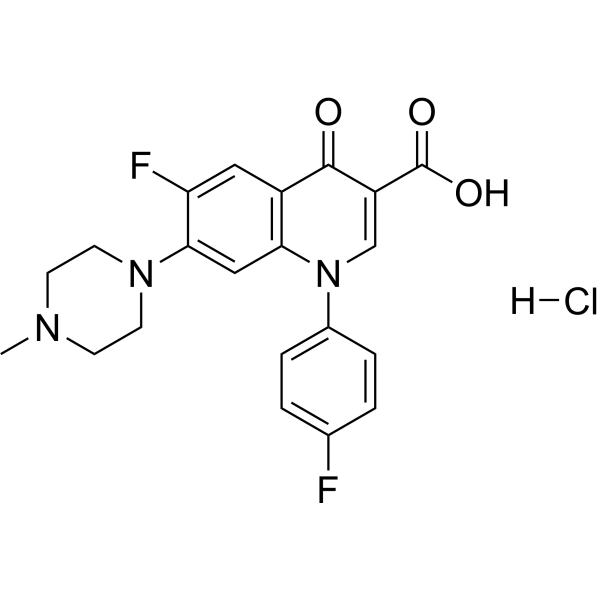 | [61] |
|  | Pazufloxacin mesylate  (Quinolone) | 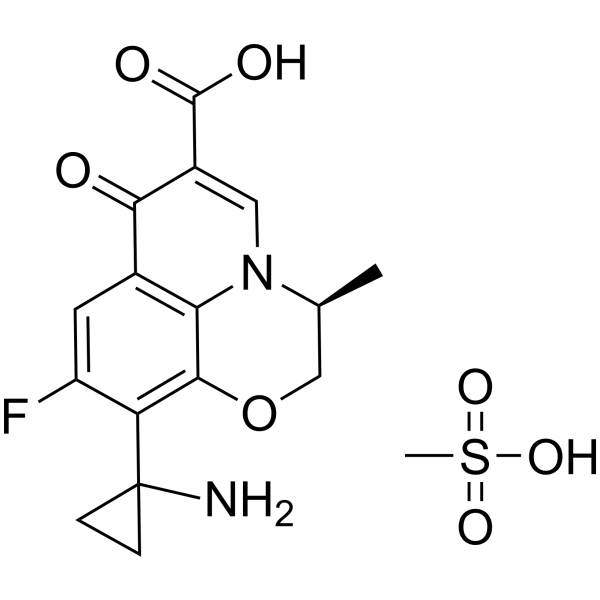 | [53] |
|  | Ozenoxacin  (Quinolone) | 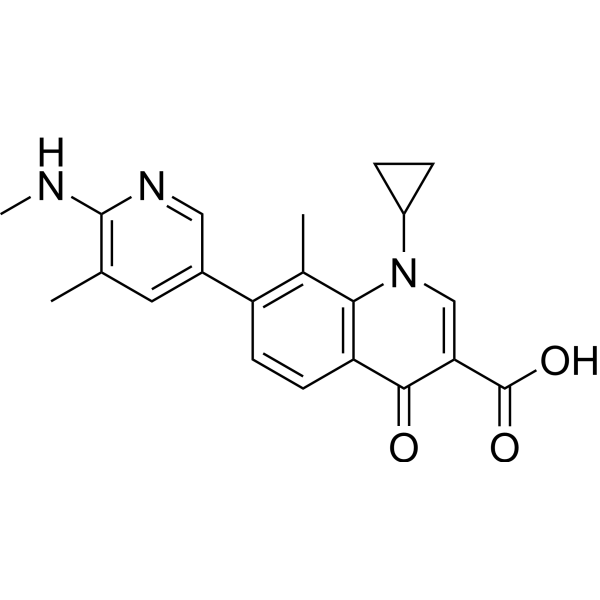 | [62] |
|  | Flumequine  (Quinolone) |  | [63] |
|  | Diiodohydroxyquinoline  (Quinolines) |  | [64] |
|  | Chlorquinaldol  (Quinolines) |  | [65] |
|  | Nitroxoline  (Quinolines) |  | [66] |
|  | Finafloxacin  (Quinolone) |  | [67] |
|  | Sarafloxacin hydrochloride  (Quinolone) |  | [41] |
|  | Garenoxacin Mesylate hydrate  (Quinolone) |  | [68] |
|  | Cadrofloxacin  (Quinolone) |  | [69] |
|  | Fluoroquinolonic acid  (Quinolone) |  | This study |
|  | Avarofloxacin  (Quinolone) |  | [70] |
|  | Danofloxacin mesylate  (Quinolone) |  | [9] |
|  | Ofloxacin  (Quinolone) |  | [50] |
|  | Norfloxacin  (Quinolone) |  | [71] |
|  | Delafloxacin meglumine  (Quinolone) |  | [72] |
|  | Oxolinic acid  (Quinolone) |  | [73] |
|  | Cadazolid  (Quinolone) |  | [9] |
|  | PBT-1033  (Quinolines) |  | [74] |
|  | Furaltadone hydrochloride  (Nitrofurans) |  | This study |
|  | Nifuratel  (Nitrofurans) |  | [75] |
|  | Furazolidone  (Nitrofurans) |  | [9] |
|  | Nifuroxazide  (Nitrofurans) |  | [9] |
|  | Nifursol  (Nitrofurans) |  | This study |
|  | Nifurpirinol  (Nitrofurans) |  | This study |
|  | Decamethoxine  (Quaternary ammonium) |  | This study |
|  | Cetylpyridinium chloride  (Quaternary ammonium) |  | [9] |
|  | Trimethyloctadecylammonium bromide  (Quaternary ammonium) |  | This study |
|  | Cetalkonium chloride  (Quaternary ammonium) |  | This study |
|  | N-Decyl-N,N-dimethyldecan-1-aminium chloride  (Quaternary ammonium) |  | This study |
|  | MBX-4132  (Oxadiazoles) |  | [76] |
|  | Mupirocin  (Medium-chain fatty acids) |  | [77] |
|  | Novobiocin sodium  (Aminocoumarin) |  | [78] |
|  | Nigericin sodium salt  (Polyether ionophore) |  | This study |
|  | Auranofin  (Gold (I)-containing compound) |  | [79] |
|  | SPR719  (Aminobenzimidazole) |  | This study |
|  | NH125  (imidazole) |  | This study |
|  | Zinc Pyrithione  (Zinc-containing compound) |  | [9] |
|  | Triclosan  (Diphenylethers) |  | [80] |
|  | Plicamycin  (oligosaccharides) |  | [9] |
|  | Chromomycin A3  (aureolic acid) |  | This study |
|  | Zoliflodacin  (Spiropyrimidinetriones) |  | [81] |
|  | Chloroxine  (Chlorobenzenes) |  | [41] |
|  | Octenidine dihydrochloride  (bispyridine) |  | [82] |
|  | Itraconazole  (triazoles) |  | [9] |
|  | Fusidic acid sodium salt  (fusidane) |  | [83] |
|  | AFN-1252  (naphthyridines) |  | [84] |
|  | Antibacterial agent 18  (multi-arm AIE) |  | This study |
|  | Walrycin B  (toxoflavin) |  | This study |
|  | Gliotoxin  (epipolythiodioxopiperazine) |  | This study |
|  | Gloxazone  (thiosemicarbazide) |  | This study |
|  | Brilacidin tetrahydrochloride  (polymer-based antibiotic) |  | [85] |

Reference

1. Fukuda D, Handa Y, Kayama Y, Fujii K, Kawamatsu S, Kawano Y, et al. The Current Landscape of Antibiotic Use and Antimicrobial Resistance in Japan: Focusing on Common Infections Including Uncomplicated Urinary Tract Infection and Gonorrhea. Antibiotics. 2025;14: 813. doi:10.3390/antibiotics14080813

2. Fujimoto K, Takemoto K, Hatano K, Nakai T, Terashita S, Matsumoto M, et al. Novel Carbapenem Antibiotics for Parenteral and Oral Applications: *In Vitro* and *In Vivo* Activities of 2-Aryl Carbapenems and Their Pharmacokinetics in Laboratory Animals. Antimicrob Agents Chemother. 2013;57: 697–707. doi:10.1128/AAC.01051-12

3. Fu KP, Foleno BD, Lafredo SC, LoCoco JM, Isaacson DM. In vitro and in vivo antibacterial activities of FK037, a novel parenteral broad-spectrum cephalosporin. Antimicrob Agents Chemother. 1993;37: 301–307. doi:10.1128/AAC.37.2.301

4. Turner JM, Connolly KL, Aberman KE, Fonseca JC, Singh A, Jerse AE, et al. Molecular Features of Cephalosporins Important for Activity against Antimicrobial-Resistant *Neisseria gonorrhoeae*. ACS Infect Dis. 2021;7: 293–308. doi:10.1021/acsinfecdis.0c00400

5. Lagacé-Wiens PRS, Adam HJ, Laing NM, Baxter MR, Martin I, Mulvey MR, et al. Antimicrobial susceptibility of clinical isolates of Neisseria gonorrhoeae to alternative antimicrobials with therapeutic potential. Journal of Antimicrobial Chemotherapy. 2017;72: 2273–2277. doi:10.1093/jac/dkx147

6. Rice RJ, Knapp JS. Susceptibility of Neisseria gonorrhoeae associated with pelvic inflammatory disease to cefoxitin, ceftriaxone, clindamycin, gentamicin, doxycycline, azithromycin, and other antimicrobial agents. Antimicrob Agents Chemother. 1994;38: 1688–1691. doi:10.1128/AAC.38.7.1688

7. Baker CN, Thornsberry C, Jones RN. In vitro antimicrobial activity of cefoperazone, cefotaxime, moxalactam (LY127935), azlocillin, mezlocillin, and other beta-lactam antibiotics against Neisseria gonorrhoeae and Haemophilus influenzae, including beta-lactamase-producing strains. Antimicrob Agents Chemother. 1980;17: 757–761. doi:10.1128/AAC.17.4.757

8. Le Saux NM, Slaney LA, Plummer FA, Ronald AR, Brunham RC. In vitro activity of ceftriaxone, cefetamet (Ro 15-8074), ceftetrame (Ro 19-5247; T-2588), and fleroxacin (Ro 23-6240; AM-833) versus Neisseria gonorrhoeae and Haemophilus ducreyi. Antimicrob Agents Chemother. 1987;31: 1153–1154. doi:10.1128/AAC.31.7.1153

9. Liang H-W. Investigating FDA-Approved Drugs for Treatment of Multidrug- Resistant Neisseria gonorrhoeae. Neisseria gonorrhoeae.

10. Piot P, Van Dyck E, Colaert J, Ursi J-P. In vitro activity of cefotaxime and other cephalosporins against Neisseria gonorrhoeae. Journal of Antimicrobial Chemotherapy. 1980;6: 47–50. doi:10.1093/jac/6.suppl_A.47

11. David R.P. Guay. Ceftaroline Fosamil&mdash;A New Broad-Spectrum Cephalosporin with Significant Activity against Methicillin-Resistant Staphylococci. Clinical Medicine Reviews in Therapeutics. 2011;3: 27–45. doi:10.4137/CMRT.S6650

12. Li X, Le W, Lou X, Genco CA, Rice PA, Su X. In Vitro Activity of Ertapenem against Neisseria gonorrhoeae Clinical Isolates with Decreased Susceptibility or Resistance to Extended-Spectrum Cephalosporins in Nanjing, China (2013 to 2019). Antimicrob Agents Chemother. 2022;66: e0010922. doi:10.1128/aac.00109-22

13. Malanoski GJ, Collins L, Wennersten C, Moellering RC, Eliopoulos GM. In vitro activity of biapenem against clinical isolates of gram-positive and gram-negative bacteria. Antimicrob Agents Chemother. 1993;37: 2009–2016. doi:10.1128/AAC.37.9.2009

14. Atia WA, Emmerson AM, Holmes D. Sultamicillin in the treatment of gonorrhoea caused by penicillin sensitive and penicillinase producing strains of Neisseria gonorrhoeae. Br J Vener Dis. 1983;59: 293–297. doi:10.1136/sti.59.5.293

15. Ng WS, Anton P, Arnold K. Neisseria gonorrhoeae strains isolated in Hong Kong: in vitro susceptibility to 13 antibiotics. Antimicrob Agents Chemother. 1981;19: 12–17. doi:10.1128/AAC.19.1.12

16. Sanchez PL, Lancaster DJ, Berg SW, Kerbs SB, Harrison WO. Cefonicid as therapy for uncomplicated gonococcal urethritis caused by penicillinase-producing Neisseria gonorrhoeae. West J Med. 1984;140: 224–226.

17. Toyosawa T, Miyazaki S, Tsuji A, Yamaguchi K, Goto S. In vitro and in vivo antibacterial activities of E1077, a novel parenteral cephalosporin. Antimicrob Agents Chemother. 1993;37: 60–66. doi:10.1128/AAC.37.1.60

18. Fuchs PC, Barry AL, Thornsberry C, Jones RN. In vitro activity of ticarcillin plus clavulanic acid against 632 clinical isolates. Antimicrob Agents Chemother. 1984;25: 392–394. doi:10.1128/AAC.25.3.392

19. Tsuji M, Ishii Y, Ohno A, Miyazaki S, Yamaguchi K. In vitro and in vivo antibacterial activities of S-1090, a new oral cephalosporin. Antimicrob Agents Chemother. 1995;39: 2544–2551. doi:10.1128/AAC.39.11.2544

20. Bharat A, Martin I, Zhanel GG, Mulvey MR. In vitro potency and combination testing of antimicrobial agents against Neisseria gonorrhoeae. Journal of Infection and Chemotherapy. 2016;22: 194–197. doi:10.1016/j.jiac.2015.10.002

21. Khan MY, Gruninger RP, Nelson SM, Obaid SR. Comparative in vitro activity of cefodizime, ceftazidime, aztreonam, and other selected antimicrobial agents against Neisseria gonorrhoeae. Antimicrob Agents Chemother. 1983;23: 477–478. doi:10.1128/AAC.23.3.477

22. López-Argüello S, Alcoceba E, Ordóñez P, Taltavull B, Cabot G, Gomis-Font MA, et al. Differential contribution of PBP occupancy and efflux on the effectiveness of β-lactams at their target site in clinical isolates of Neisseria gonorrhoeae. Balcazar JL, editor. PLoS Pathog. 2024;20: e1012783. doi:10.1371/journal.ppat.1012783

23. Zheng X-L, Xu W-Q, Liu J-W, Zhu X-Y, Chen S-C, Han Y, et al. Evaluation of Drugs with Therapeutic Potential for Susceptibility of Neisseria Gonorrhoeae Isolates from 8 Provinces in China from 2018. IDR. 2020;Volume 13: 4475–4486. doi:10.2147/IDR.S278020

24. Khan MY, Siddiqui Y, Simpson ML, Gruninger RP. Comparative in vitro activity of cefmenoxime, cefotaxime, cefuroxime, cefoxitin, and penicillin against Neisseria gonorrhoeae. Antimicrob Agents Chemother. 1981;20: 681–682. doi:10.1128/AAC.20.5.681

25. Murphy SP, Erwin ME, Jones RN. Cefquinome (HR 111V) in vitro evaluation of a broad-spectrum cephalosporin indicated for infections in animals. Diagnostic Microbiology and Infectious Disease. 1994;20: 49–55. doi:10.1016/0732-8893(94)90019-1

26. Jones RN, Critchley IA, Whittington WLH, Janjic N, Pottumarthy S. Activity of faropenem tested against Neisseria gonorrhoeae isolates including fluoroquinolone-resistant strains. Diagnostic Microbiology and Infectious Disease. 2005;53: 311–317. doi:10.1016/j.diagmicrobio.2005.06.014

27. Barbee LA, Golden MR. Aztreonam for Neisseria gonorrhoeae: a systematic review and meta-analysis. Journal of Antimicrobial Chemotherapy. 2020;75: 1685–1688. doi:10.1093/jac/dkaa108

28. Sinha RK, Rosenthal RS. Effect of penicillin G on release of peptidoglycan fragments by Neisseria gonorrhoeae: characterization of extracellular products. Antimicrob Agents Chemother. 1981;20: 98–103. doi:10.1128/AAC.20.1.98

29. Jones RN, Barry AL. Antimicrobial activity, spectrum, and recommendations for disk diffusion susceptibility testing of ceftibuten (7432-S; SCH 39720), a new orally administered cephalosporin. Antimicrob Agents Chemother. 1988;32: 1576–1582. doi:10.1128/AAC.32.10.1576

30. Vázquez JA, Martín E, Galarza P, Giménez MJ, Aguilar L, Coronel P. In vitro susceptibility of Spanish isolates of Neisseria gonorrhoeae to cefditoren and five other antimicrobial agents. International Journal of Antimicrobial Agents. 2007;29: 473–474. doi:10.1016/j.ijantimicag.2006.10.014

31. Connolly KL, Eakin AE, Gomez C, Osborn BL, Unemo M, Jerse AE. Pharmacokinetic Data Are Predictive of *In Vivo* Efficacy for Cefixime and Ceftriaxone against Susceptible and Resistant *Neisseria gonorrhoeae* Strains in the Gonorrhea Mouse Model. Antimicrob Agents Chemother. 2019;63: e01644-18. doi:10.1128/AAC.01644-18

32. Ochiai S, Ishiko H, Yasuda M, Deguchi T. Rapid detection of the mosaic structure of the Neisseria gonorrhoeae penA Gene, which is associated with decreased susceptibilities to oral cephalosporins. J Clin Microbiol. 2008;46: 1804–1810. doi:10.1128/JCM.01800-07

33. Ito M, Yasuda M, Yokoi S, Ito S, Takahashi Y, Ishihara S, et al. Remarkable increase in central Japan in 2001-2002 of Neisseria gonorrhoeae isolates with decreased susceptibility to penicillin, tetracycline, oral cephalosporins, and fluoroquinolones. Antimicrob Agents Chemother. 2004;48: 3185–3187. doi:10.1128/AAC.48.8.3185-3187.2004

34. Mehaffey PC, Putnam SD, Barrett MS, Jones RN. Evaluation of in vitro spectra of activity of azithromycin, clarithromycin, and erythromycin tested against strains of Neisseria gonorrhoeae by reference agar dilution, disk diffusion, and Etest methods. J Clin Microbiol. 1996;34: 479–481. doi:10.1128/jcm.34.2.479-481.1996

35. Hardy DJ, Hensey DM, Beyer JM, Vojtko C, McDonald EJ, Fernandes PB. Comparative in vitro activities of new 14-, 15-, and 16-membered macrolides. Antimicrob Agents Chemother. 1988;32: 1710–1719. doi:10.1128/AAC.32.11.1710

36. Thayer JD, Field FW, Perry MI, Martin JE, Garson W. Surveillance studies on Neisseria gonorrhoea sensitivity to penicillin and nine other antibiotics. Bull World Health Organ. 1961;24: 327–331.

37. Unemo M, Golparian D, Sánchez-Busó L, Grad Y, Jacobsson S, Ohnishi M, et al. The novel 2016 WHO *Neisseria gonorrhoeae* reference strains for global quality assurance of laboratory investigations: phenotypic, genetic and reference genome characterization. J Antimicrob Chemother. 2016;71: 3096–3108. doi:10.1093/jac/dkw288

38. Biddle JW, Thornsberry C. In vitro activity of rosamicin, josamycin, erythromycin, and clindamycin against beta-lactamase-nagative and beta-lactamase-positive strains of Neisseria gonorrhoeae. Antimicrob Agents Chemother. 1979;15: 243–245. doi:10.1128/AAC.15.2.243

39. Golparian D, Fernandes P, Ohnishi M, Jensen JS, Unemo M. *In Vitro* Activity of the New Fluoroketolide Solithromycin (CEM-101) against a Large Collection of Clinical Neisseria gonorrhoeae Isolates and International Reference Strains, Including Those with High-Level Antimicrobial Resistance: Potential Treatment Option for Gonorrhea? Antimicrob Agents Chemother. 2012;56: 2739–2742. doi:10.1128/AAC.00036-12

40. Thabaut A, Meyran M, Huerre M. In-vitro comparison of macrolides, lincosamides and synergistins on Neisseria gonorrhoeae. J Antimicrob Chemother. 1985;16 Suppl A: 213–215. doi:10.1093/jac/16.suppl_a.213

41. Foerster S, Gustafsson TN, Brochado AR, Desilvestro V, Typas A, Unemo M. The first wide‐scale drug repurposing screen using the Prestwick Chemical Library (1200 bioactive molecules) against *Neisseria gonorrhoeae* identifies high *in vitro* activity of auranofin and many additional drugs. APMIS. 2020;128: 242–250. doi:10.1111/apm.13014

42. Tupasi TE, Crisologo LB, Torres CA, Calubiran OV, de Jesus I. Cefuroxime, thiamphenicol, spectinomycin, and penicillin G in uncomplicated infections due to penicillinase-producing strains of Neisseria gonorrhoeae. Br J Vener Dis. 1983;59: 172–175. doi:10.1136/sti.59.3.172

43. Fujii K, Tsuji A, Miyazaki S, Yamaguchi K, Goto S. In vitro and in vivo antibacterial activities of KRM-1648 and KRM-1657, new rifamycin derivatives. Antimicrob Agents Chemother. 1994;38: 1118–1122. doi:10.1128/AAC.38.5.1118

44. Duzer J van, Michaelis A, Geiss W, Stafford D, Raker J, Yu X, et al. Rifamycin analogs and uses thereof. US20050197333A1, 2005. Available: https://patents.google.com/patent/US20050197333A1/en

45. Nazli A, He DL, Xu H, Wang Z-P, He Y. A Comparative Insight on the Newly Emerging Rifamycins: Rifametane, Rifalazil, TNP-2092 and TNP-2198. Curr Med Chem. 2022;29: 2846–2862. doi:10.2174/0929867328666210806114949

46. FINLAND M, GARNER C, WILCOX C, SABATH LD. Susceptibility of Neisseria gonorrhoeae to 66 Antibacterial Agents in Vitro. Journal of the American Venereal Disease Association. 1976;2: 33–40.

47. Lee H, Kim H, Seo YH, Yong D, Jeong SH, Lee K, et al. In vitro activity of tigecycline alone and antimicrobial combinations against clinical Neisseria gonorrhoeae isolates. Diagnostic Microbiology and Infectious Disease. 2017;87: 160–162. doi:10.1016/j.diagmicrobio.2016.10.022

48. Segreti J, Hirsch DJ, Harris AA, Kapell KS, Orbach H, Kessler HA. In vitro activity of tosufloxacin (A-61827; T-3262) against selected genital pathogens. Antimicrob Agents Chemother. 1990;34: 971–973. doi:10.1128/AAC.34.6.971

49. Talbot H, Romanowski B. In vitro activities of lomefloxacin, tetracycline, penicillin, spectinomycin, and ceftriaxone against Neisseria gonorrhoeae and Chlamydia trachomatis. Antimicrob Agents Chemother. 1989;33: 2049–2051. doi:10.1128/AAC.33.12.2049

50. Carlyn CJ, Doyle LJ, Knapp CC, Ludwig MD, Washington JA. Activities of three investigational fluoroquinolones (BAY y 3118, DU-6859a, and clinafloxacin) against Neisseria gonorrhoeae isolates with diminished susceptibilities to ciprofloxacin and ofloxacin. Antimicrob Agents Chemother. 1995;39: 1606–1608. doi:10.1128/AAC.39.7.1606

51. Wise R, Andrews JM, Matthews R, Wolstenholme M. The in-vitro activity of two new quinolones: rufloxacin and MF 961. J Antimicrob Chemother. 1992;29: 649–660. doi:10.1093/jac/29.6.649

52. Jönsson A, Foerster S, Golparian D, Hamasuna R, Jacobsson S, Lindberg M, et al. In vitro activity and time-kill curve analysis of sitafloxacin against a global panel of antimicrobial-resistant and multidrug-resistant Neisseria gonorrhoeae isolates. APMIS. 2018;126: 29–37. doi:10.1111/apm.12777

53. Tanaka M, Nakayama H, Haraoka M, Saika T, Kobayashi I, Naito S. Susceptibilities of Neisseria gonorrhoeae isolates containing amino acid substitutions in GyrA, with or without substitutions in ParC, to newer fluoroquinolones and other antibiotics. Antimicrob Agents Chemother. 2000;44: 192–195. doi:10.1128/AAC.44.1.192-195.2000

54. Jones RN, Biedenbach DJ, Ambrose PG, Wikler MA. Zabofloxacin (DW-224a) activity against Neisseria gonorrhoeae including quinolone-resistant strains. Diagnostic Microbiology and Infectious Disease. 2008;62: 110–112. doi:10.1016/j.diagmicrobio.2008.05.010

55. Hook EW, Pinson GB, Blalock CJ, Johnson RB. Dose-ranging study of CP-99,219 (trovafloxacin) for treatment of uncomplicated gonorrhea. Antimicrob Agents Chemother. 1996;40: 1720–1721. doi:10.1128/AAC.40.7.1720

56. Dobson RA, O’Connor JR, Poulin SA, Kundsin RB, Smith TF, Came PE. In vitro antimicrobial activity of rosoxacin against Neisseria gonorrhoeae, Chlamydia trachomatis, and Ureaplasma urealyticum. Antimicrob Agents Chemother. 1980;18: 738–740. doi:10.1128/AAC.18.5.738

57. Berrón S, Vázquez JA, Giménez MJ, de la Fuente L, Aguilar L. In vitro susceptibilities of 400 Spanish isolates of Neisseria gonorrhoeae to gemifloxacin and 11 other antimicrobial agents. Antimicrob Agents Chemother. 2000;44: 2543–2544. doi:10.1128/AAC.44.9.2543-2544.2000

58. Van Der Willigen AH, Van Der Hoek JC, Wagenvoort JH, Van Vliet HJ, Van Klingeren B, Schalla WO, et al. Comparative double-blind study of 200- and 400-mg enoxacin given orally in the treatment of acute uncomplicated urethral gonorrhea in males. Antimicrob Agents Chemother. 1987;31: 535–538. doi:10.1128/AAC.31.4.535

59. Thapa E, Knauss HM, Colvin BA, Fischer BA, Weyand NJ. Persistence Dynamics of Antimicrobial-Resistant Neisseria in the Pharynx of Rhesus Macaques. Antimicrob Agents Chemother. 2020;64: e02232-19. doi:10.1128/AAC.02232-19

60. Biedenbach DJ, Beach ML, Jones RN. Antimicrobial activity of gatifloxacin tested against Neisseria gonorrhoeae using three methods and a collection of fluoroquinolone-resistant strains. Diagnostic Microbiology and Infectious Disease. 1998;32: 307–311. doi:10.1016/S0732-8893(98)00114-X

61. Bowie WR, Shaw CE, Chan DG, Boyd J, Black WA. In vitro activity of difloxacin hydrochloride (A-56619), A-56620, and cefixime (CL 284,635; FK 027) against selected genital pathogens. Antimicrob Agents Chemother. 1986;30: 590–593. doi:10.1128/AAC.30.4.590

62. Morrissey I, Cantón R, Vila J, Gargallo-Viola D, Zsolt I, Garcia-Castillo M, et al. Microbiological Profile of Ozenoxacin. Future Microbiol. 2019;14: 773–787. doi:10.2217/fmb-2019-0089

63. Rohlfing SR, Landmesser JE, Gerster JF, Pecore SE, Stern RM. Differentiation of fluorinated quinolone antibacterials with Neisseria gonorrhoeae isolates. J Antimicrob Chemother. 1985;15: 539–544. doi:10.1093/jac/15.5.539

64. Mensah E, Fourie PB, Peters RPH. Antimicrobial effects of Medicines for Malaria Venture Pathogen Box compounds on strains of *Neisseria gonorrhoeae*. Odom John A, editor. Antimicrob Agents Chemother. 2023;67: e00348-23. doi:10.1128/aac.00348-23

65. Corrihons I, Dutilh B, Bébéar C. [In vitro activity of an antiseptic, chlorquinaldol, against Neisseria gonorrhoeae and Chlamydia trachomatis]. Pathol Biol (Paris). 1991;39: 136–139.

66. Fuchs F, Wille J, Hamprecht A, Parcina M, Lehmann C, Schwarze-Zander C, et al. In vitro activity of mecillinam and nitroxoline against Neisseria gonorrhoeae – re-purposing old antibiotics in the multi-drug resistance era. Journal of Medical Microbiology. 2019;68: 991–995. doi:10.1099/jmm.0.001014

67. Stubbings W, Leow P, Yong GC, Goh F, Körber-Irrgang B, Kresken M, et al. In vitro spectrum of activity of finafloxacin, a novel, pH-activated fluoroquinolone, under standard and acidic conditions. Antimicrob Agents Chemother. 2011;55: 4394–4397. doi:10.1128/AAC.00833-10

68. Deshpande L, Biedenbach DJ, Jones RN. Antimicrobial activity of BMS 284756 (T-3811) against Neisseria gonorrhoeae tested by three methods. International Journal of Antimicrobial Agents. 2001;18: 437–440. doi:10.1016/S0924-8579(01)00438-1

69. Cottarel G, Gardner TS, Lei X, Porco J, Schaus SE, Wierzbowski J, et al. Compositions and methods for potentiating antibiotic activity. US20100234348A1, 2010. Available: https://patents.google.com/patent/US20100234348A1/en

70. Biedenbach DJ, Turner LL, Jones RN, Farrell DJ. Activity of JNJ-Q2, a novel fluoroquinolone, tested against Neisseria gonorrhoeae, including ciprofloxacin-resistant strains. Diagnostic Microbiology and Infectious Disease. 2012;74: 204–206. doi:10.1016/j.diagmicrobio.2012.06.006

71. Tanaka M, Otsuki M, Nishino T, Kobayashi I, Matsumoto T, Kumazawa J. Mutation in DNA gyrase of norfloxacin-resistant clinical isolates of Neisseria gonorrhoeae. Genitourin Med. 1996;72: 295–297. doi:10.1136/sti.72.4.295

72. Soge OO, Salipante SJ, No D, Duffy E, Roberts MC. In Vitro Activity of Delafloxacin against Clinical Neisseria gonorrhoeae Isolates and Selection of Gonococcal Delafloxacin Resistance. Antimicrob Agents Chemother. 2016;60: 3106–3111. doi:10.1128/AAC.02798-15

73. Barry AL, Jones RN, Thornsberry C, Ayers LW, Gerlach EH, Sommers HM. Antibacterial activities of ciprofloxacin, norfloxacin, oxolinic acid, cinoxacin, and nalidixic acid. Antimicrob Agents Chemother. 1984;25: 633–637. doi:10.1128/AAC.25.5.633

74. Jen FE-C, Edwards JL, El-Deeb IM, Walker MJ, von Itzstein M, Jennings MP. Repurposing the Ionophore, PBT2, for Treatment of Multidrug-Resistant Neisseria gonorrhoeae Infections. Antimicrob Agents Chemother. 2022;66: e0231821. doi:10.1128/aac.02318-21

75. Churcher GM, Evans AJ. Inhibition of Neisseria gonorrhoeae by nifuratel. Br J Vener Dis. 1969;45: 149–150. doi:10.1136/sti.45.2.149

76. Aron ZD, Mehrani A, Hoffer ED, Connolly KL, Srinivas P, Torhan MC, et al. trans-Translation inhibitors bind to a novel site on the ribosome and clear Neisseria gonorrhoeae in vivo. Nat Commun. 2021;12: 1799. doi:10.1038/s41467-021-22012-7

77. Cern A, Connolly KL, Jerse AE, Barenholz Y. In Vitro Susceptibility of Neisseria gonorrhoeae Strains to Mupirocin, an Antibiotic Reformulated for Parenteral Administration in Nanoliposomes. Antimicrob Agents Chemother. 2018;62: e02377-17. doi:10.1128/AAC.02377-17

78. Ishizaki Y, Hayashi C, Matoba K, Igarashi M. Novobiocin primarily targets ParE in Neisseria gonorrhoeae. J Antibiot. 2025;78: 159–165. doi:10.1038/s41429-024-00797-1

79. Elkashif A, Seleem MN. Investigation of auranofin and gold-containing analogues antibacterial activity against multidrug-resistant Neisseria gonorrhoeae. Sci Rep. 2020;10: 5602. doi:10.1038/s41598-020-62696-3

80. Chitsaz M, Booth L, Blyth MT, O’Mara ML, Brown MH. Multidrug Resistance in Neisseria gonorrhoeae: Identification of Functionally Important Residues in the MtrD Efflux Protein. mBio. 2019;10: e02277-19. doi:10.1128/mBio.02277-19

81. Unemo M, Ahlstrand J, Sánchez-Busó L, Day M, Aanensen D, Golparian D, et al. High susceptibility to zoliflodacin and conserved target (GyrB) for zoliflodacin among 1209 consecutive clinical *Neisseria gonorrhoeae* isolates from 25 European countries, 2018. Journal of Antimicrobial Chemotherapy. 2021;76: 1221–1228. doi:10.1093/jac/dkab024

82. Lopez K. Antimicrobial Strategies for Topical Applications. Doctor of Philosophy, Louisiana State University and Agricultural and Mechanical College. 2020. doi:10.31390/gradschool_dissertations.5304

83. Jones RN, Biedenbach DJ, Roblin PM, Kohlhoff SA, Hammerschlag MR. Update on fusidic acid (CEM-102) tested against Neisseria gonorrhoeae and Chlamydia trachomatis. Antimicrob Agents Chemother. 2010;54: 4518–4519. doi:10.1128/AAC.00235-10

84. Yao J, Bruhn DF, Frank MW, Lee RE, Rock CO. Activation of Exogenous Fatty Acids to Acyl-Acyl Carrier Protein Cannot Bypass FabI Inhibition in Neisseria. Journal of Biological Chemistry. 2016;291: 171–181. doi:10.1074/jbc.M115.699462

85. Abdelsattar AS, Abutaleb NS, Seleem MN. A novel peptide mimetic, brilacidin, for combating multidrug-resistant Neisseria gonorrhoeae. Rahman AS, editor. PLoS One. 2025;20: e0325722. doi:10.1371/journal.pone.0325722
