# Supplementary material for: Screening a library of antibacterial compounds leads to discovery of novel inhibitors for Neisseria gonorrhoeae and Chlamydia trachomatis
Source: PLoS One. 2026 Feb 6;21(2):e0340486. doi: 10.1371/journal.pone.0340486 (PMC12880645; doi:10.1371/journal.pone.0340486)
Supplement: Attachment — Submitted filename: Point-by-point response to reviwers.docx [file pone.0340486.s003.docx]

December 16, 2025

Dear Dr. Yung-Fu Chang,

Please find enclosed a revised version of manuscript **ID: PONE-D-25-61453** entitled “**Screening a library of antibacterial compounds leads to discovery of novel inhibitors for *Neisseria gonorrhoeae* and *Chlamydia trachomatis***” by Abdallah S. Abdelsattar, Nader S. Abutaleb, and Mohamed N. Seleem.

We thank you and the reviewers for carefully reviewing our manuscript and providing valuable comments to improve the manuscript. We have addressed the points raised by the reviewers and provided the clarifying comments below.

We would like to have this revised manuscript considered for publication in **PLOS ONE**.

Thank you very much.

Yours sincerely,

Mohamed Seleem, Ph.D.

Tyler and Frances Young Endowed Chair of Bacteriology

Professor, Biomedical Sciences and Pathobiology

Center for One Health Research, Virginia Tech

1410 Prices Fork RD, Blacksburg, VA, 24061

**Reviewer #1:**

This manuscript effectively addresses the critical unmet need for a single therapeutic agent against co-infections of Chlamydia trachomatis and Neisseria gonorrhoeae. Utilizing a drug repurposing approach, the authors screened an antibacterial compound library and successfully identified gloxazone and SPR719 as promising dual-action agents, demonstrating potent activity against both pathogens. The findings are highly significant as they propose immediate candidates for further preclinical development to combat the growing crisis of antibiotic-resistant sexually transmitted infections (STIs). Given the quality of the data and the clinical importance of the findings, the manuscript is well-suited for publication with minor revisions. My detailed comments are below:

1. Abstract: I assume the 211 million is the number of cases worldwide. Please specify it.
1. **Response:** Thank you for this comment. Yes, it is worldwide, so we added the word “worldwide” to specify the number.
2. Method: In the section describing the MIC determination against N. gonorrhoeae, please specify what broth medium was used.
2. **Response:** We added that the media was “Brucella broth supplemented with 1% IsoVitaleX.”
3. Result: 1. The first paragraph of the results section doesn’t fit in there because it only mentions anti- N. gonorrhoeae therapeutics. Please consider deleting it or re-writing it.
3. **Response:** Based on your recommendation, we deleted the first paragraph of the results section.
4. Result: 2. The description of the Immunofluorescence images is insufficient. It is currently difficult to interpret the outcome of the compound treatment from the images alone. Please add a more detailed description explaining what the key markers/colors represent and how the compound treatment visibly alters the morphology or life cycle of C. trachomatis. Also, please clarify if a series of concentrations was tested for the IF images to correlate with the MIC determination.
4. **Response:** Thank you. We added a paragraph in the results section and modified the legend to explain the color code. “The addition of SPR719, gloxazone, or azithromycin at a fixed concentration (1 μM) completely blocked the development of *C. trachomatis* inclusion bodies. However, the negative control (DMSO) shows the mature inclusions after 48 hours. Inclusion bodies were identified using primary antibodies bound to *Chlamydia* MOMPs and a secondary antibody conjugated to Alexa 488, which appears as green circles (Fig. 2). ”
5. Result: 3. Given that N. gonorrhoeae is known to potentially induce a difficult-to-treat persistence-like state in C. trachomatis, it is highly relevant to the goal of finding a single co-infection therapeutic. Have you tested the inhibitory effects of your hit compounds (gloxazone and SPR719) on both N. gonorrhoeae and C. trachomatis simultaneously in a co-culture assay? If not, this should be discussed as a future direction.
5. **Response:** Thank you for this comment, and we added a future direction in the last paragraph: “We recommend further in vitro studies to assess the inhibitory effects of hit compounds (gloxazone and SPR719) on both N. gonorrhoeae and C. trachomatis simultaneously in a co-culture assay.”
6. Reference: Please ensure that the format of the references is consistent throughout the entire Reference section.
6. **Response:** We confirmed the references are consistent.
